# Cognitive Workload and Mental Burden in Health Care Professionals Interacting With AI: Systematic Review and Meta-Analysis

**DOI:** 10.2196/93618

**Published:** 2026-08-04

**Authors:** Eun Jeong Gong, Chang Seok Bang, Jae Jun Lee

**Affiliations:** 1 Institute for Liver and Digestive Diseases, Hallym University, Chuncheon, Republic of Korea; 2 Institute of New Frontier Research, Hallym University College of Medicine, Chuncheon, Republic of Korea; 3 Department of Internal Medicine, Hallym University College of Medicine, Sakju-ro 77, Chuncheon, 24253, Republic of Korea, 82 82 33 240 582; 4 Department of Anesthesiology and Pain Medicine, Hallym University College of Medicine, Chuncheon, Republic of Korea

**Keywords:** artificial intelligence, cognitive workload, mental fatigue, human-AI interaction, alert fatigue, verification burden

## Abstract

**Background:**

AI adoption in health care has accelerated rapidly, with ambient documentation tools, diagnostic imaging AI, and clinical decision support systems (CDSSs) entering routine practice. However, the cognitive demands placed on clinicians supervising these systems remain understudied. Specifically, the concept of verification burden requires closer examination. Consequently, institutional decision-makers lack a structured, certainty-graded evidence base regarding the true impact of AI on clinician workload and burnout.

**Objective:**

This study aimed to systematically review evidence on cognitive workload and burnout in health care professionals that use AI-powered clinical tools, quantify pooled effects under a conservative inferential framework, and assess certainty of evidence by AI category.

**Methods:**

The study was registered in PROSPERO (CRD420261284298) and reported per PRISMA 2020 and PRISMA-S guidelines. We searched MEDLINE, Embase, Web of Science, and Cochrane CENTRAL (January 2015-2026) for studies measuring cognitive workload or burnout using validated instruments (NASA Task Load Index [NASA-TLX] and Professional Fulfillment Index [PFI]) among health care professionals using clinical AI. Risk of bias was assessed using ROB 2.0 and ROBINS-I; certainty was rated using GRADE. Meta-analyses applied Hartung-Knapp-Sidik-Jonkman adjustment with restricted maximum likelihood estimation, incorporating prediction intervals (PIs).

**Results:**

We included 21 studies representing 2885 health care professionals across 7 countries. The synthesis demonstrated that the cognitive impact of clinical AI varies according to its specific application. Pooled analyses of ambient AI documentation showed statistically significant reductions in NASA-TLX temporal demand (SMD −1.46, 95% CI −2.81 to −0.11; k=2; *I*^2^=31.1%) and effort (SMD −1.29, 95% CI −2.16 to −0.42; k=2; *I*^2^=0%), PFI work exhaustion (MD −0.35, 95% CI −0.58 to −0.12; k=3; *I*^2^=0%; 95% PI −1.03 to 0.33), and burnout prevalence (OR 0.47, 95% CI 0.25-0.86; k=3; *I*^2^=0%; 95% PI 0.06-3.82). Two pools favored ambient AI but did not reach significance at k=2: NASA-TLX mental demand (SMD −1.29, 95% CI −3.64 to 1.07) and documentation time (SMD −0.24, 95% CI −1.10 to 0.61). Diagnostic imaging AI and CDSS showed mixed or paradoxically increased workload. GRADE certainty was moderate for cognitive workload reduction with ambient AI, low for burnout reduction with ambient AI, and very low for imaging AI and CDSS outcomes.

**Conclusions:**

This review combines validated workload instruments, meta-analysis, and PIs in health care AI, delivering a GRADE certainty assessment across 5 AI categories that prior accuracy- or efficiency-focused reviews have not provided. Ambient AI documentation was associated with reduced cognitive workload and burnout, but only in voluntary early-adopter cohorts and based on few studies; the conservative CIs were wide and, where estimable, PIs crossed the null. Findings inform institutional pilots with prospective workload measurement, regulatory human-factors evaluation of AI medical devices, and human-centered AI design. Net benefit on the health care workforce remains an open empirical question.

## Introduction

The rapid adoption of artificial intelligence (AI) in health care is changing clinical workflows across various medical specialties. From diagnostic imaging analysis to ambient documentation scribes, AI-powered tools are increasingly integrated into routine clinical practice. According to the American Medical Association, physician AI use nearly doubled from 38% in 2023 to 66% in 2024, reflecting rapid integration compared to historical health care technology adoptions [1].

These tools are intended to alleviate the administrative burden that has been identified as a primary driver of clinician burnout. This clinician crisis peaked at 62.8% prevalence in 2021, though rates have since declined to approximately 45% as of 2023 [2]. Algorithmic solutions are considered practical largely because they can automate cognitively demanding tasks, particularly clinical documentation. A landmark time-motion study demonstrated that physicians spend 49.2% of their office day on electronic health record and desk work combined, with only 27% on direct clinical face time, and an additional 1‐2 hours of electronic health record work each evening [3]. Early evidence from ambient AI scribes suggests meaningful reductions in documentation time, and these tools are frequently promoted to reduce administrative burden [4]. However, it remains unclear whether these systems reduce overall cognitive burden or shift it from content generation to verification.

Recent commercial deployments in 2024 and 2025 have accelerated this enthusiasm. Tierney et al [5] reported substantial reductions in documentation burden following an enterprise-wide rollout of an ambient AI scribe to over 3000 clinicians, and Albrecht et al [6] described similar quality-improvement gains in a multispecialty implementation. These 2 enterprise deployments illustrate the pace of commercial scaling but do not contribute outcome data to this review because neither used a validated cognitive-workload instrument, such as the National Aeronautics and Space Administration Task Load Index (NASA-TLX), or the Physician Task Load Index, or a validated burnout instrument as a prespecified primary outcome. Consequently, neither met the eligibility criteria (detailed in the Methods section). Throughout this text, commercial deployment data, theoretical frameworks, and prior reviews provide background only; the primary, secondary, and exploratory outcomes reported in the Results section derive solely from the 21 studies that met the eligibility criteria.

Subsequent trials and observational studies reported reductions in NASA-TLX subscales, Professional Fulfillment Index (PFI) work exhaustion, and burnout prevalence among adopters of ambient AI documentation systems [7-10]. However, this rapidly growing literature is concentrated on a small number of commercial products (predominantly Abridge and Dragon Ambient Experience [DAX] Copilot) deployed in voluntary early-adopter cohorts, mostly in US outpatient primary care, with follow-up periods rarely exceeding 3 months. The rapid pace of commercial deployment has outpaced the accumulation of independent, multiproduct, long-term human-factors evidence. This disparity motivates a systematic quantitative synthesis of existing data to map current empirical gaps.

Integrating AI into clinical workflows modifies the clinician’s role from an active creator of clinical content to a supervisor of AI-generated outputs. This transition imposes novel cognitive demands that differ qualitatively from traditional documentation tasks. Clinicians must engage in continuous verification of AI recommendations with clinical judgment, a phenomenon characterized as verification burden. Human factors research suggests that such monitoring tasks are cognitively demanding in ways that human cognitive architecture is not well suited to sustain, particularly given the ease with which grammatically polished AI-generated text may bypass critical evaluation [11]. Conceptually, verification burden differs from extraneous cognitive load, which is a construct from cognitive load theory describing task-irrelevant demands imposed by suboptimal interface or instructional design. Instead, verification burden is inherent to the AI-supervision task itself, aligning closely with Bainbridge’s “ironies of automation” framework [12], where automation generates monitoring demands that may exceed the cognitive savings provided.

Theoretical frameworks from automation science provide important context for understanding this relationship. Bainbridge’s [12] work on the ironies of automation described how systems designed to reduce human workload often create new cognitive demands through the requirement for vigilance and oversight. The out-of-the-loop performance problem further suggests that operators monitoring automated systems experience degraded situation awareness and diminished capacity to detect errors [13]. Applied to health care AI, these frameworks predict that clinicians may experience automation complacency, defined as a reduced tendency to verify AI outputs, leading to missed errors despite sustained cognitive effort. These constructs, including verification burden, automation bias, and automation complacency, were not measured by validated instruments in any included study; they are framed throughout as a hypothesis-generating interpretive lens [14].

Despite the proliferation of health care AI tools and concern about their human factors implications, empirical evidence on cognitive workload remains sparse. A 2024 systematic review examining AI implementation in medical imaging found only 3 studies addressing clinician workload, noting that no study assessed workload separately in terms of cognitive workload changes, and describing this gap as remarkable [15]. Similarly, while numerous studies have examined AI’s impact on documentation time, few have used validated instruments to measure the subjective cognitive experience of clinicians interacting with these systems. Only a small subset of ambient AI scribe studies uses validated cognitive workload instruments such as the NASA-TLX; most rely instead on documentation time, single-item satisfaction ratings, or burnout scales as proxies. Studies of diagnostic imaging AI and alert-based clinical decision support systems (CDSSs) have likewise not consistently measured cognitive experience. For instance, a prospective evaluation of computer-aided detection (CADe) for prostate magnetic resonance imaging (MRI) found no reduction in radiologist workload despite improved diagnostic performance [16], and a usability evaluation of a pediatric sepsis prediction model documented increased perceived workload and alert fatigue rather than the expected reduction [17].

The heterogeneity of AI applications, outcome measures, and study designs has limited previous attempts at a quantitative synthesis with explicit certainty ratings. Consequently, institutional decision-makers and regulatory authorities lack a structured evidence base to guide implementation. This gap is significant for patient safety and clinician well-being, given that cognitive workload is a predictor of medical errors, burnout, and workforce attrition [18]. If AI tools reduce physical documentation effort while imposing equivalent or greater cognitive demands through verification requirements, the net benefit for clinicians may be minimal or even negative. Understanding this trade-off is essential for evidence-based AI implementation and the design of human-centered clinical AI systems. This systematic review aims to synthesize the available evidence on cognitive workload and mental burden experienced by health care professionals when interacting with AI-powered clinical tools. Specifically, we sought (1) to quantify cognitive workload associated with AI-assisted clinical tasks using validated instruments, (2) to identify factors that influence cognitive burden across different AI applications and clinical domains, and (3) to evaluate related constructs including burnout, automation bias, and verification burden that may contribute to clinicians’ psychological strain when working with AI systems.

## Methods

### Protocol and Registration

This systematic review and meta-analysis was conducted in accordance with the PRISMA (Preferred Reporting Items for Systematic Reviews and Meta-Analyses) 2020 expanded checklist [19] and the PRISMA-S (Preferred Reporting Items for Systematic Reviews and Meta-Analyses Literature Search Extension) extension for search reporting [20]. Completed reporting checklists are provided as Checklist 1 (PRISMA 2020 for Abstracts), Checklist 2 (PRISMA 2020 expanded checklist), and Checklist 3 (PRISMA-S extension), with each item mapped to the corresponding manuscript location. The protocol was prospectively registered with the International Prospective Register of Systematic Reviews (PROSPERO; CRD420261284298, registered on January 13, 2026). Patients or the public were not involved in the design, conduct, reporting, or dissemination plans of our research.

### Eligibility Criteria

Eligibility was determined using the population, intervention, comparator, and outcomes framework [21]. Eligible populations included health care professionals actively engaged in clinical practice or clinical research, whereas medical students and nonclinical administrative staff were excluded. Eligible interventions consisted of AI-powered clinical tools, such as ambient AI documentation systems, CDSS, diagnostic AI, large language models (LLMs), predictive AI, triage systems, and AI-based burnout intervention applications. The comparator included traditional workflows without AI assistance, preimplementation periods, or no comparator for single-arm studies.

Primary outcomes focused on cognitive workload, mental fatigue, and burnout, provided they were measured with validated instruments. Acceptable cognitive workload measures included the NASA-TLX [22], Subjective Workload Assessment Technique [23], or Paas Cognitive Load Scale [24]. Burnout had to be evaluated using established tools such as the Maslach Burnout Inventory (MBI) [25], Oldenburg Burnout Inventory (OLBI) [26], Stanford PFI [27], Copenhagen Burnout Inventory (CBI) [28], or Mini-Z [29]. Secondary outcomes included alert fatigue, automation bias, trust calibration, verification burden, and System Usability Scale [30].

Eligible study designs included randomized controlled trials (RCTs), non-RCTs, prospective and retrospective cohort studies, cross-sectional studies, and pre- and postimplementation studies. While all eligible studies entered the qualitative narrative synthesis, meta-analytic pooling was deliberately restricted to within-AI-category subsets to avoid combining functionally heterogeneous tools. Consequently, all 6 prespecified meta-analytic pools were drawn from the ambient AI documentation subset. Studies evaluating diagnostic imaging AI, CDSS, LLM inbox tools, and AI-based burnout interventions were reported in the narrative synthesis only and were not pooled together. Qualitative-only studies, editorials, commentaries, conference abstracts without full text, and systematic reviews were excluded. Studies measuring only time-based efficiency outcomes without validated workload instruments, studies assessing only diagnostic accuracy, and those evaluating nonclinical AI applications were also excluded.

### Information Sources

Our systematic search covered 4 electronic databases spanning January 2015 to January 2026: MEDLINE (via PubMed), Embase (via OVID), Cochrane CENTRAL, and Web of Science Core Collection. Additional sources included manual searching of reference lists, forward citation tracking, and screening of preprint servers (medRxiv and arXiv) for recent studies not yet indexed in bibliographic databases.

### Search Strategy

The search strategy combined 3 concepts using Boolean operators: AI and clinical AI tools, health care professionals, and cognitive workload or burnout. The complete database-specific search strategies are provided in Multimedia Appendix 1.

### Selection Process

Search results were imported into EndNote (version 21; Clarivate Analytics) and deduplicated. Two reviewers (CSB and EJG) independently screened titles and abstracts against eligibility criteria, followed by an independent full-text assessment. Disagreements were resolved through discussion or by consulting a third reviewer (JJL) if consensus could not be reached. The selection process was documented using a PRISMA 2020 flow diagram.

### Data Collection Process and Data Items

Data were extracted independently by 2 reviewers using a standardized extraction form. For studies with multiple intervention groups or time points, data from all relevant arms were extracted. Authors were contacted for clarification or additional data when necessary. The extracted information included study characteristics (authors, year, country, journal, and study design), population characteristics (sample size, participant type, clinical specialty, and experience level), intervention details (AI system type, clinical domain, and implementation setting), outcome measures (validated instruments used, outcome definitions, and measurement time points), and results (effect estimates, CIs, *P* values, and pre-post comparisons).

### Study Risk of Bias Assessment

Risk of bias was assessed independently by 2 reviewers using domain-appropriate tools. For RCTs, we used the Cochrane Risk of Bias tool version 2.0 (RoB 2.0) [31], which evaluates bias arising from the randomization process, deviations from intended interventions, missing outcome data, measurement of the outcome, and selection of reported results. For non-RCTs, we used the Risk of Bias in Non-randomized Studies of Interventions (ROBINS-I) tool [32], which evaluates bias due to confounding, selection of participants, classification of interventions, deviations from intended interventions, missing data, measurement of outcomes, and selection of reported results. Each domain was rated as “low risk,” “some concerns,” or “high risk” for RoB 2.0, and “low,” “moderate,” “serious,” or “critical” risk for ROBINS-I. Disagreements were resolved by discussion.

### Effect Measures

We calculated standardized mean differences (SMDs and Hedges *g*) for continuous outcomes measured with different scales (eg, NASA-TLX subscales using 0‐10 vs 0‐20 ranges). For continuous outcomes measured with the same instrument, mean differences (MDs) were used. Odds ratios (ORs) were calculated for binary outcomes (eg, burnout prevalence). For pre-post studies, a within-individual correlation of *r*=0.7 was assumed between baseline and follow-up, accompanied by sensitivity analyses were performed at *r*=0.5 and *r*=0.9.

### Synthesis Methods

We adopted a narrative synthesis as the primary integrative approach due to anticipated heterogeneity across AI applications, clinical domains, outcome measures, and study designs [33]. Where 2 or more studies reported the same outcome using the same validated instrument in comparable contexts, random-effects meta-analysis was performed. No participant overlap exists across the 6 prespecified meta-analytic pools. NASA-TLX data were pooled on a subscale-by-subscale basis (mental demand, temporal demand, and effort) using Hedges *g* to standardize different scale ranges (0‐10 and 0‐20). We explicitly avoided pooling aggregate scores from modified versions of the instrument, combining only studies that reported identical subscales on comparable measurement structures. This pooling strategy introduces measurement heterogeneity, which is addressed in the Limitations section. Statistical heterogeneity was assessed using *I*^2^ (<25%, 25%‐75%, >75% for low, moderate, and high, respectively), complemented by τ^2^. Subgroup analyses were planned by study design, health care professional type, AI system type, and geographic region; however, the small number of studies per outcome precluded these analyses.

### Statistical Synthesis Methods

Random-effects meta-analyses applied the Hartung-Knapp-Sidik-Jonkman (HKSJ) adjustment [34] uniformly as the primary inferential method for all 6 prespecified pools, irrespective of the number of contributing studies. The between-study variance τ^2^ was estimated by restricted maximum likelihood. We constructed Knapp-Hartung CIs using the 2-tailed *t* distribution. The variance-inflation factor q* was truncated to a minimum of 1 to ensure the adjusted interval remained at least as wide as the unadjusted random-effects CIs [34]. Prediction intervals (PIs) were calculated for pools containing 3 or more studies [35,36]. For pools with only 2 studies, we reported the pooled point estimate with its 95% Knapp-Hartung CI without a PI. This conservative approach intentionally trades narrow-band precision for protection against type I error inflation.

CIs are used to quantify the precision of the average pooled effect, whereas PIs estimate the expected distribution of true effects across new settings. These metrics are reported alongside each other where estimable. We confirmed that no participants were shared between studies in any meta-analytic pool. The NASA-TLX subscale pools [4,10], the PFI work exhaustion pool [7,8,37], the burnout prevalence pool [4,9,38], and the documentation time pool [10,39] all combine nonoverlapping samples; therefore, no correlation adjustment for shared participants was required. Where pre-post designs contributed continuous outcomes, we calculated effect sizes using change-score SDs derived from pooled pre and post SDs assuming a pre-post correlation of *r*=0.7 with sensitivity analyses at *r*=0.5 and *r*=0.9. For burnout prevalence, included studies [4,9,38] reported paired pre-post counts within the same cohort, and paired ORs were computed (or derived from pooled pre or post 2×2 tables where individual-level data were unavailable). This paired approach is more conservative than treating proportions as independent because it accounts for within-individual correlation.

### Reporting Bias Assessment

We did not perform formal assessments of reporting bias using funnel plots or Egger test because all pools contained fewer than the recommended threshold of 10 studies [40]. Instead, the potential for small-study effects was considered qualitatively within the GRADE (Grading of Recommendations Assessment, Development and Evaluation) assessment.

### Certainty Assessment

The certainty of evidence for each outcome was assessed using the GRADE approach [41]. Evidence was rated as high, moderate, low, or very low based on 5 domains: risk of bias, inconsistency, indirectness, imprecision, and publication bias. GRADE-Confidence in the Evidence from Reviews of Qualitative Research for qualitative findings was prespecified in the protocol but could not be performed because no qualitative-only study was identified.

### Reporting Standards and Protocol Deviations

Reporting standards and the corresponding completed checklists are described in Checklists 1-3. The protocol deviations described in this section reflect refinements made during the conduct of the review and are reported transparently in keeping with PRISMA 2020 item 5. The systematic review was prospectively registered in PROSPERO prior to data extraction (CRD420261284298, registered on January 13, 2026).

We acknowledge the following protocol deviations. First, a lower-bound search date restriction of January 2015 was applied to focus on the contemporary clinical AI era. Second, medical students were excluded because their clinical exposure to AI tools differs substantially from that of practicing clinicians. Third, qualitative-only studies were excluded, which consequently omitted the planned thematic synthesis and GRADE-Confidence in the Evidence from Reviews of Qualitative Research assessments. Fourth, we additionally searched Cochrane CENTRAL to capture trials potentially unindexed elsewhere. Fifth, burnout was elevated to a coprimary outcome due to its prevalence in the identified literature. Sixth, several prespecified secondary outcomes were omitted because no included studies measured them with validated instruments. Seventh, AI-based burnout intervention applications were retained, as they directly addressed the research question. Finally, the HKSJ adjustment was uniformly adopted as the primary inferential method for all meta-analytic pools, replacing the DerSimonian-Laird estimator.

## Results

### Study Selection

**Figure 1.**
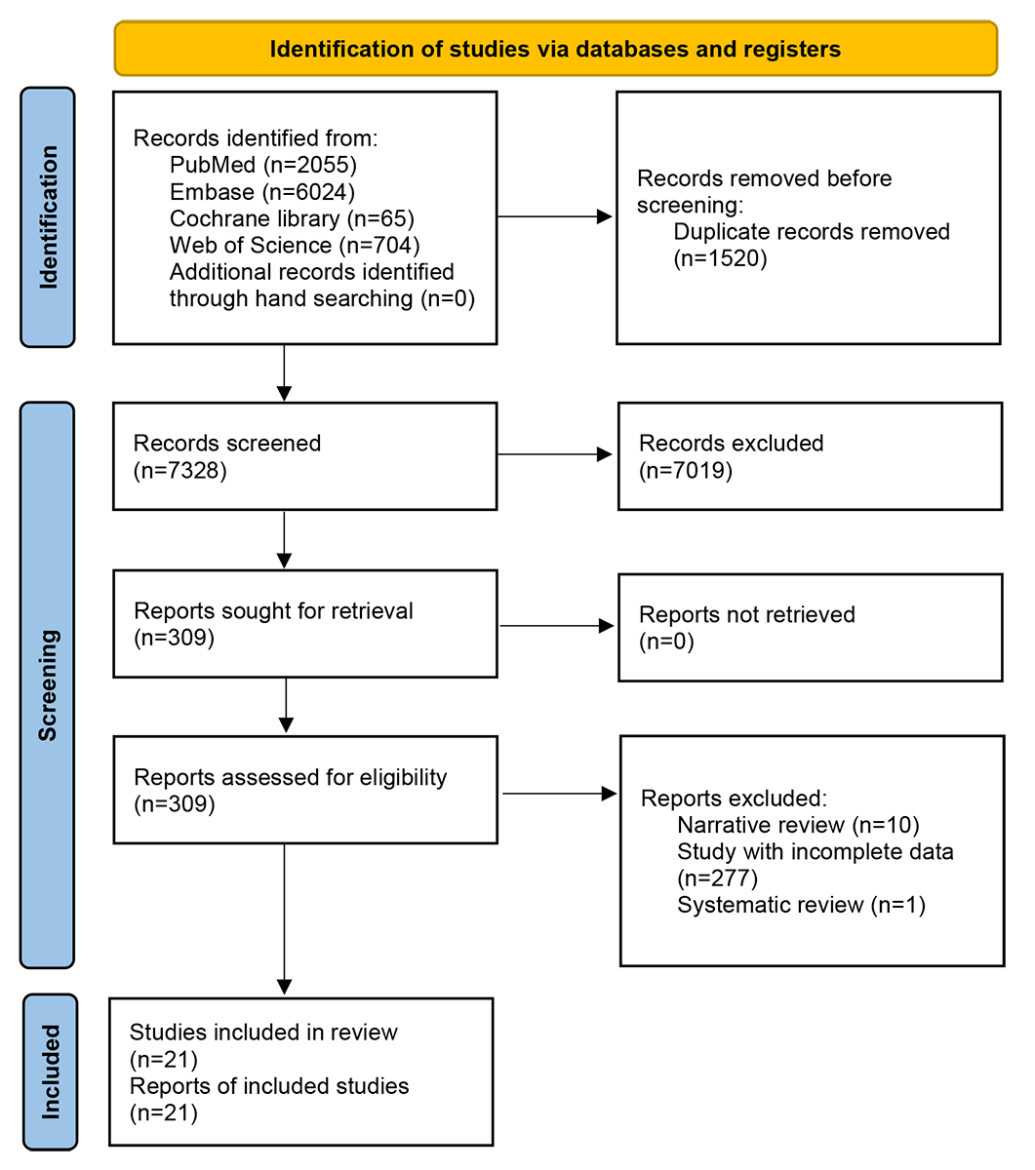
PRISMA (Preferred Reporting Items for Systematic Reviews and Meta-Analyses) 2020 flow diagram of the study selection process.

The systematic search identified 8848 records across 4 databases: PubMed or MEDLINE (n=2055), Embase or Ovid (n=6024), Cochrane CENTRAL (n=65), and Web of Science (n=704). After deduplication in EndNote, 1520 duplicate records were removed. This left 7328 unique records that underwent title or abstract screening, of which 7019 records were excluded. The remaining 309 reports were retrieved and assessed for full-text eligibility. We excluded 288 papers for specific reasons, including narrative review (n=10), study with incomplete data (n=277), and systematic review (n=1). Furthermore, we excluded 2 large ambient AI scribe studies because they did not meet the validated instrument criterion. Specifically, Tierney et al [5] (n=7260) used custom satisfaction surveys without NASA-TLX or standardized burnout measures, and Albrecht et al [6] (n=181) relied on a custom quality improvement survey without validated workload assessments. In total, 21 studies met all inclusion criteria and were included in the qualitative synthesis. The study selection process is presented in Figure 1.

### Study Characteristics

The 21 included studies [4,7-10,16,17,37-39,42-52] were published between 2019 and 2025. In total, 19 (90.5%) of these studies [4,7-10,16,17,39-45,48-52] were published in 2024‐2025, indicating recent expansion in this research area. Studies were conducted across 7 countries: the United States (15/21, 71.4%) [4,7-10,17,37-39,42-45,47,49], Germany (1/21, 4.8%) [16], Australia (1/21, 4.8%) [50], South Korea (1/21, 4.8%) [51], the United Kingdom (1/21, 4.8%) [46], Zambia (1/21, 4.8%) [48], and the United Arab Emirates (1/21, 4.8%) [52]. The total number of health care professionals across all studies was 2885, with sample sizes ranging from 7 to 1430 participants.

Regarding study design, 3 studies were RCTs [7,8,51], which consisted of 1 parallel 3-arm RCT [7], 1 stepped-wedge RCT [8], and 1 single-blind 3-group RCT [51]. In total, 3 further studies used randomized crossover designs [43,50,52], 2 were randomized simulation studies [46,49], and 13 were observational studies [4,9,10,16,17,37-39,42,44,45,47,48]. The observational group included pre-post designs (n=9) [9,10,16,17,37-39,42,45], cross-sectional studies (n=2) [44,47], quality improvement studies (n=1) [4], and mixed methods study (n=1) [48].

Participants included physicians (n=19) [4,7-10,16,17,37-39,42-47,49,50,52], advanced practice providers (APPs; n=9) [4,8-10,38,39,43-45], nurses (n=3) [17,50,51], and radiology trainees (n=2) [47,48]. The evaluated clinical domains covered outpatient documentation (n=12) [4,7-10,37-39,42-45], diagnostic imaging (n=3) [16,47,48], pediatric clinical care (n=4) [17,38,47,49], trauma and transfusion (n=1) [50], inpatient documentation (n=1) [46], nursing practice (n=1) [51], and psychiatric practice (n=1) [52].

AI interventions were categorized into 5 types: ambient AI documentation systems (n=13) [4,7-10,38,39,42-46,52], CDSS (n=3) [17,49,50], radiology AI (n=3) [16,47,48], LLM-based inbox management (n=1) [37], and AI-based burnout intervention (n=1) [51]. The characteristics of included studies are summarized in Table 1.

**Table 1.** Characteristics of the 21 included studies.

Study	Country	Study design	Setting	Values, n	Participants	AI^a^ system	Instruments	Follow-up duration	Primary outcome	Key findings	Risk of bias	Interpretation
Ambient AI documentation (n=13)												
Lukac et al (2025) [7]	United States	Parallel 3-arm pragmatic RCT^b^	Multispecialty clinic (outpatient)	238	Physicians	DAX^c^ Copilot; Nabla	Mini-Z 2.0, 4-item PTL^d^, PFI^e^-WE^f^	12 weeks	Task load, WE	PTL: DAX −39.9, Nabla −31.7 (*P*<.01); PFI-WE −0.27 (0‐4 scale; *P*=.01); documentation time: Nabla −9.5%	Low	Ambient AI significantly reduces physician task load across multiple specialties
Afshar et al (2025) [8]	United States	Stepped-wedge RCT	Primary care (outpatient)	66	Physicians, APPs^g^	Abridge	Stanford PFI, PDQI-9^h^	24 weeks	WE	WE/ID^i^: −0.44 (95% CI −0.62 to −0.25; *P*<.001)	Some concerns	Reduction in burnout with sustained 6-month follow-up
Olson et al (2025) [4]	United States	Multicenter quality improvement pre-post	Multisite (outpatient)	263	Physicians, APPs	Abridge	Single-item burnout, 3-item NASA-TLX^j^	3‐6 months	Burnout prevalence	Burnout: 51.9%→38.8% (aOR^k^ 0.26; *P*<.001); TLX^l^: −2.64	Serious	13 pp^n^ absolute burnout reduction, single-arm pre- and postdesign precludes causal or scalability claims
Shah et al (2025) [42]	United States	Prospective quality improvement pre-post	Academic medical center (outpatient)	48	Physicians	DAX Copilot	NASA-TLX, SUS^m^, PFI	8 weeks	Cognitive workload	NASA-TLX: −24.42 (*P*<.001); burnout: −1.94; SUS: +10.9	Serious	Large NASA-TLX reduction (24 points) observed, with high usability; uncontrolled pre- and postdesign
Hudson et al (2025) [43]	United States	Crossover RCT	Academic medical center (outpatient)	40	Physicians, APPs	Abridge	NASA-TLX	2 weeks	Cognitive load	NASA-TLX: 221→118 (−60.7%; *P*<.001); mental −57%	Moderate	60% reduction represents largest effect observed
You et al (2025) [9]	United States	Multisite pre-post	Academic medical center (outpatient)	1430	Physicians, APPs	Multiple ambient AI systems	Stanford PFI	42 days	Burnout, well-being	Burnout: −21.2 pp^n^ (*P*<.001)	Serious	Largest sample size; 21 pp reduction at 42 days in the MGB^o^ cohort; no control group
Owens et al (2024) [44]	United States	Cross-sectional	Primary care	110	PCPs^p^	DAX	OLBI^q^	—^r^	Burnout	Disengagement: −2.1 (*P*<.05)	Serious	Higher AI use associated with lower disengagement in cross-sectional comparison; causal direction cannot be inferred
Misurac et al (2025) [45]	United States	Pre-post	Academic medical center (outpatient)	35	Physicians, APPs	Ambient AI (not specified)	Stanford PFI	3 months	Burnout	Burnout: 69%→43% (*P*=.005)	Serious	26 pp absolute burnout reduction in an uncontrolled pre- and postsample (n=35)
Stults et al (2025) [10]	United States	Pre-post	Integrated health system (outpatient)	100	Ambulatory clinicians	Abridge	NASA-TLX, single-item burnout	12 weeks	Documentation time, workload	Time: 6.2→5.3 minutes (*P*<.001); NASA-TLX mental demand ↓; burnout: 42.1%→35.1%	Serious	Multisite replication of ambient AI benefits; 7 pp burnout reduction consistent with other studies
Duggan et al (2025) [39]	United States	Prospective quality improvement pre-post	Academic medical center (outpatient)	46	Physicians, APPs	DAX Copilot	NASA-TLX items, SUS	8 weeks	Documentation burden, efficiency	SUS positive; documentation burden perceived ↓; mixed individual experiences	Serious	Highlights variability in clinician experience; not all users benefit equally from AI scribes
Pelletier et al (2025) [38]	United States	Pre-post with ITSA^s^	Pediatric hospital (outpatient)	84	Pediatric physicians, APPs	Abridge	NASA-TLX, Mini-Z	6 months	Documentation time, workload, burnout	Time: −2.8 minutes per appointment (*P*<.001); 1.5 hours per week saved; NASA-TLX ↓; Mini-Z burnout ↓	Moderate	First pediatric ambient AI study; ITSA strengthens causal inference; pediatric settings
Bracken et al (2026) [46]	United Kingdom	Simulation	Simulated inpatient ward (orthopedic surgery)	7	PGY^t^-1 doctors	Heidi Health	NASA-TLX, PDQI-9	1 session	Documentation time	Time: 27 versus 128 seconds; frustration −79%	Critical	Large simulated time savings; n=7, simulation only—critical risk of bias
Nawaz et al (2025) [52]	United Arab Emirates	Crossover simulation	Psychiatric hospital (simulation)	8	Psychiatrists	Lyrebird Health	NASA-TLX, SAIL^u^	1 session	Workload, documentation quality	NASA-TLX: 25.0 versus 461.3 (*P*<.001); largest effect in review; SAIL quality ↑	Critical	Preprint; largest effect in this review but n=8, simulation only—critical risk of bias; first psychiatric ambient AI study
Diagnostic imaging AI (n=3**)**												
Wenderott et al (2024) [16]	Germany	Prospective pre-post	Academic medical center	91 cases	Radiologists	Prostate MRI^v^ CADe^w^ (commercial, not specified)	NASA-TLX, STAI^x^	6 months	Workload, reading time	NASA-TLX: NS^y^ (*P*=.51); time ↑15.7→23.1 minutes (*P*=.02)	Moderate	Critical negative finding: diagnostic AI may increase rather than decrease cognitive demands
Lopez-Rippe et al (2025) [47]	United States	Mixed methods	Academic medical center	NR^z^	Radiology trainees	RADHawk	NASA-TLX	6 months	Cognitive load	Reduced workload and mental demand (values NR)	Moderate	Knowledge-based AI support may reduce trainee burden, but quantitative data lacking
Muzumala et al (2025) [48]	Zambia	Comparative experiment	Pediatric hospital	12	Radiology residents	Pneumonia chest x-ray diagnosis AI (not specified)	NASA-TLX, TAM^aa^-2	1 session	Workload, usability	NASA-TLX: 1.86 (7-point scale); positive TAM-2	Serious	Low-resource setting feasibility demonstrated; modified scale limits cross-study comparison
CDSS^ab^ (n=3)												
Richardson et al (2019) [49]	United States	Crossover simulation	Pediatric hospital	32	Pediatric physicians	PedsGuide (Children’s Mercy)	NASA-TLX, SUS	1 session	Mental workload	Mental: 6.34 versus 11.8 (*P*<.001); SUS: 88/100	Low	Well-designed CDSS can halve mental demand while improving clinical performance
Kandaswamy et al (2025) [17]	United States	Mixed methods	Pediatric emergency department	40	Clinicians, nurses	IPSO^ac^ sepsis AI (CHOA^ad^)	NASA-TLX, SUS	6 months	Workload, trust	NASA-TLX: 43→57 (workload ↑); trust: 3.8/5	Moderate	Alert-based AI paradoxically increases workload; highlights verification burden and alert fatigue
Sanderson et al (2023) [50]	Australia	Crossover simulation	Pediatric hospital	44	Physicians, nurses	MT^ae^-CDS^af^ (Westmead Hospital)	NASA-TLX, SUS	1 session	Workload, decisions	NASA-TLX: CDS 57.1 versus paper 64.5 (*P*=.005); SUS: 82.5	Low	CDS reduces workload in high-stakes decisions while improving decision velocity
Large language model (LLM)–based tools (n=1)												
Garcia et al (2024) [37]	United States	Prospective quality improvement pre-post	Hospital	162	Physicians	GPT-4 (OpenAI)	PTL, PFI-WE	5 weeks	Task load	PTL: 61.3→47.3 (−13.9; *P*<.001); WE: −0.33	Moderate	Early LLM evidence shows promise for inbox management; 20% adoption rate indicates acceptability
AI burnout intervention (n=1)												
Baek and Cha (2025) [51]	Korea	3-group RCT	Hospital	120	Nurses	Nurse Healing Space (AI burnout application)	CBI^ag^	4 weeks	Burnout	Client burnout: 62.6→42.0 (*P*=.001); personal: 67.5→44.7	Low	AI-tailored intervention reduced nursing burnout in an RCT; zero dropout

a AI: artificial intelligence.

b RCT: randomized controlled trial.

c DAX: Dragon Ambient Experience.

d PTL: Physician Task Load.

e PFI: Professional Fulfillment Index.

f WE: Work Exhaustion.

g APP: advanced practice provider.

h PDQI-9: Physician Documentation Quality Instrument.

i ID: interpersonal disengagement.

j NASA-TLX: National Aeronautics and Space Administration Task Load Index.

k aOR: adjusted odds ratio.

l TLX: Task Load Index.

m SUS: System Usability Scale.

n pp: percentage point.

o MGB: Mass General Brigham.

p PCP: primary care physician.

q OLBI: Oldenburg Burnout Inventory.

r Not available.

s ITSA: interrupted Time Series Analysis.

t PGY: postgraduate year.

u SAIL: Sheffield Assessment Instrument for Letters.

v MRI: magnetic resonance imaging.

w CADe: computer-aided detection.

x STAI: State-Trait Anxiety Inventory.

y NS: not significant.

z NR: not reported.

aa TAM: Technology Acceptance Model.

ab CDSS: clinical decision support system.

ac IPSO: Improving Pediatric Sepsis Outcomes.

ad CHOA: Children’s Healthcare of Atlanta.

ae MT: massive transfusion.

af CDS: clinical decision support.

ag CBI: Copenhagen Burnout Inventory.

### Validated Instruments Used

The NASA-TLX or its derivative instruments were used in 16 (76%) studies [4,7,10,16,17,37-39,42,43,46-50,52]. These applications included the full 6-subscale version [16,17,42,46,49,50,52] or abbreviated versions, such as the 4-item Physician Task Load [7,37] and the 3-item NASA-TLX [4,10,39,43]. Pelletier et al [38] used the NASA-TLX without specifying subscale details. Burnout-specific instruments included the Stanford PFI (n=5) [7-9,42,45], Mini-Z (n=3) [7,10,38], OLBI (n=1) [44], CBI (n=1) [51], and single-item burnout measures (n=2) [4,10]. No study used the full MBI despite its status as the most widely validated burnout measure [25]. The System Usability Scale was used as a secondary outcome in 5 studies [37,39,42,49,50], and the Sheffield Assessment Instrument for Letters was used in 1 study to assess documentation quality [52].

### Risk of Bias Assessment

Risk of bias varied substantially across studies. Among the RCTs, the 3-arm RCT by Lukac et al [7] was rated as having “low risk” across all domains. The stepped-wedge RCT by Afshar et al [8] was rated as having “some concerns” due to potential period effects and lack of blinding inherent to the intervention. The 3-group RCT by Baek and Cha [51] was rated as “low risk” with zero dropouts and appropriate randomization.

**Figure 2.**
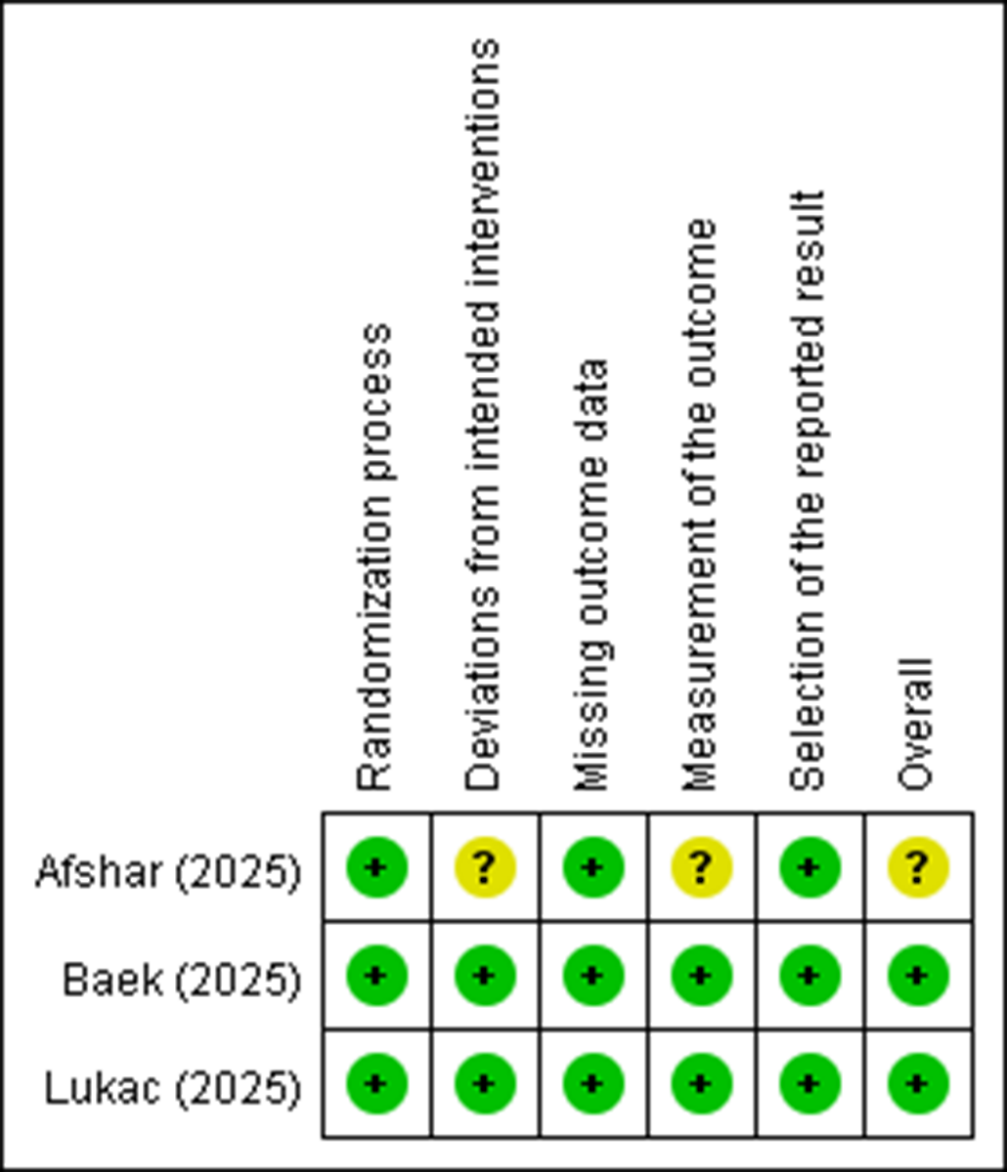
Risk-of-bias summary for the 3 randomized controlled trials assessed with the Cochrane Risk of Bias tool version 2.0 (RoB 2) [7,8,51]. Green plus signs denote low risk of bias, and yellow question marks denote some concerns across the 5 RoB 2.0 domains and the overall judgment. Detailed domain-level judgments with supporting rationale are reported in Table 2.

Among non-RCTs assessed using ROBINS-I [32], 2 studies were rated as “low” risk of bias [49,50], 6 were rated as “moderate” risk of bias [16,17,37,38,43,47], 8 were rated as “serious” risk primarily due to confounding and selection bias [4,9,10,39,42,44,45,48], and 2 simulation studies were rated as “critical” risk due to small sample sizes (n=7 and n=8, respectively) [46,52]. Common methodological limitations included lack of control groups, short follow-up periods, voluntary participation introducing selection bias, and use of abbreviated or modified versions of validated instruments without separate validation. The risk of bias assessment is presented in Figure 2 and Tables 2 and 3.

**Table 2.** Risk of bias assessment using the Cochrane Risk of Bias tool version 2.0 for the 3 included randomized controlled trials.

Study	D1: randomization	D2: deviations from interventions	D3: missing outcome data	D4: measurement of outcome	D5: selection of reported result	Overall	Direction
Lukac et al (2025) [7]	Low (adequate 1:1:1 allocation, concealment)	Low (protocol followed)	Low (<5% dropout)	Low (objective EHR^a^+ validated PROs^b^)	Low (preregistered)	Low	—^c^
Afshar et al (2025) [8]	Low (stepped-wedge appropriate)	Some concerns (no blinding, period effects possible)	Low (adequate retention)	Some concerns (self-reported, unblinded)	Low (all prespecified outcomes reported)	Some concerns	Favors intervention
Baek and Cha (2025) [51]	Low (appropriate randomization)	Low (single-blind, participants blinded)	Low (0 dropouts)	Low (validated CBI^d^)	Low (all outcomes reported)	Low	—

a EHR: electronic health record.

b PRO: patient-reported outcome.

c Not available.

d CBI: Copenhagen Burnout Inventory.

**Table 3.** Risk of bias assessment using ROBINS-I^a^ for the 18 nonrandomized studies.

Study	D1: Confounding	D2: Selection	D3: Classification	D4: Deviations	D5: Missing data	D6: Measurement	D7: Selection of results	Overall
Olson et al (2025) [4]	Serious (no control, multisite confounders)	Moderate (voluntary early adopters)	Low (clear AI^b^ vs no AI)	Moderate (variable adoption rates across sites)	Low (complete EHR^c^ data)	Low (validated instruments)	Low (all outcomes reported)	Serious
Shah et al (2025) [42]	Serious (no control, pre-post)	Moderate (single site, technology-savvy population)	Low (clear intervention)	Low (structured implementation)	Low (adequate completion)	Low (validated instruments)	Low (transparent reporting)	Serious
Hudson et al (2025) [43]	Moderate (crossover controls within-subject confounding)	Moderate (single site)	Low (clear intervention)	Low (standardized protocol)	Low (complete data)	Low (validated instruments)	Low (all outcomes reported)	Moderate
You et al (2025) [9]	Serious (no control, secular trends)	Moderate (single academic center)	Low (clear AI exposure)	Low (consistent implementation)	Moderate (62% survey response rate)	Low (validated instruments)	Low (complete reporting)	Serious
Owens et al (2024) [44]	Serious (no control, QI^d^ design)	Serious (self-selected champions, early adopters)	Low (clear intervention)	Low (protocol followed)	Low (adequate retention)	Moderate (mixed validated or unvalidated items)	Low (all outcomes reported)	Serious
Misurac et al (2025) [45]	Serious (no control, QI study)	Moderate (single institution)	Low (clear AI use documented)	Low (standardized rollout)	Low (complete EHR metrics)	Moderate (abbreviated NASA-TLX^e^ without separate validation)	Low (transparent reporting)	Serious
Bracken et al (2026) [46]	Critical (simulation not real practice, artificial conditions)	Critical (n=7 junior doctors only, selection bias)	Low (clear intervention)	Low (controlled simulation)	Low (complete data)	Low (full NASA-TLX)	Low (all outcomes reported)	Critical
Stults et al (2025) [10]	Serious (no control, pre-post QI)	Moderate (purposively sampled champions, Sutter Health investor in Abridge)	Low (clear Abridge use)	Low (structured 12-week implementation)	Low (57% survey, 92% EHR data)	Low (self-reported NASA-TLX subscales)	Low (all outcomes reported)	Serious
Duggan et al (2025) [39]	Serious (no control, pre-post)	Serious (voluntary enrollment, n=46 from 17 specialties, heterogeneous)	Low (clear DAX^f^ Copilot use)	Low (8-week structured pilot)	Moderate (incomplete survey responses noted)	Moderate (self-reported SUS^g^ and NASA-TLX items)	Low (mixed results transparently reported)	Serious
Pelletier et al (2025) [38]	Moderate (ITSA^h^ controls secular trends, level+ slope analysis)	Moderate (broader recruitment n=84, multiple pediatric specialties)	Low (clear Abridge use)	Low (6-month structured implementation)	Low (complete EHR metrics)	Moderate (mixed objective EHR +subjective surveys)	Low (all outcomes reported)	Moderate
Nawaz et al (2025) [52]	Critical (simulation environment, not real clinical practice)	Critical (n=8 only, selection bias)	Low (clear Lyrebird use)	Low (controlled crossover protocol)	Low (complete data, small n)	Moderate (simulation may artificially inflate effects)	Low (all outcomes reported)	Critical
Wenderott et al (2024) [16]	Moderate (within-subject comparison partially controls confounding)	Moderate (single radiology department)	Low (clear AI vs no AI cases)	Low (standardized reading protocol)	Low (complete case data)	Low (validated NASA-TLX)	Low (all outcomes reported)	Moderate
Lopez-Rippe et al (2025) [47]	Moderate (crossover design reduces confounding)	Serious (convenience sample of radiology trainees)	Low (clear AI intervention)	Low (structured simulation)	Moderate (incomplete workload assessments)	Moderate (abbreviated NASA-TLX)	Low (transparent reporting)	Moderate
Muzumala et al (2025) [48]	Serious (no control, observational only)	Moderate (single site, limited generalizability)	Low (clear AI use)	Low (consistent implementation)	Low (adequate data capture)	Serious (custom workload measure, not validated NASA-TLX)	Low (all outcomes reported)	Serious
Richardson et al (2019) [49]	Low (randomized crossover simulation controls confounding)	Low (adequate sample n=32)	Low (clear CDSS^i^ intervention)	Low (standardized protocol)	Low (complete data)	Low (full NASA-TLX, validated)	Low (all outcomes reported)	Low
Kandaswamy et al (2025) [17]	Moderate (pre-post with some statistical adjustment)	Moderate (multisite pediatric units)	Low (clear sepsis AI exposure)	Low (structured implementation)	Low (complete outcome data)	Low (validated NASA-TLX)	Low (all outcomes reported)	Moderate
Sanderson et al (2023) [50]	Low (randomized crossover design)	Low (adequate sample n=44, diverse participants)	Low (clear CDSS intervention)	Low (standardized trauma scenarios)	Low (complete data)	Low (full NASA-TLX)	Low (all outcomes reported)	Low
Garcia et al (2024) [37]	Moderate (pre-post without control, novel intervention)	Moderate (single academic site, early adopters)	Low (clear LLM^j^ inbox tool use)	Low (consistent implementation)	Moderate (30% nonresponse to follow-up survey)	Low (validated instruments)	Low (all outcomes reported)	Moderate

a ROBINS-I: Risk of Bias in Non-randomized Studies of Interventions.

b AI: artificial intelligence.

c EHR: electronic health record.

d QI: quality improvement.

e NASA-TLX: National Aeronautics and Space Administration Task Load Index.

f DAX: Dragon Ambient Experience.

g SUS: System Usability Scale.

h ITSA: interrupted time series analysis.

i CDSS: clinical decision support system.

j LLM: large language model.

### Certainty of Evidence

The certainty of evidence was assessed using the GRADE approach for each outcome-intervention combination (Table 4). The certainty of evidence for cognitive workload reduction with ambient AI documentation was rated as moderate (⊕⊕⊕◯). Evidence from 13 studies (2 RCTs and 11 observational or quasi-experimental) [4,7-10,38,39,42-46,52] consistently demonstrated NASA-TLX reductions of 24-40 points. The evidence was not downgraded for risk of bias, as the 2 RCTs showed low risk and observational studies showed consistent direction of effect. No serious inconsistency, indirectness, or imprecision was identified. Because every pool contained fewer than 10 studies, formal small-study-effect testing was not performed. Consequently, the possibility of small-study effects was considered qualitatively and judged not to warrant downgrading for this outcome.

**Table 4.** GRADE (Grading of Recommendations Assessment, Development and Evaluation) summary of findings for the 6 prespecified meta-analytic pools (uniform Knapp-Hartung adjustment, restricted maximum likelihood estimation of τ^2^).

Question	Studies, n	Patients, n	Certainty assessment						Effect		Certainty
			Study design	Risk of bias	Inconsistency	Indirectness	Imprecision	Other considerations	Relative (95% CI)	Absolute (95% CI)	
Ambient AI^a^ documentation compared to control in NASA-TLX^b^ mental demand	2	308	Nonrandomized studies	Serious^c^	Serious^d^	Not serious	Serious^e^	None	—^f^	SMD^g^ 1.29 SD lower (3.64 lower to 1.07 higher)	⨁◯◯◯ Very low^c,d,e^
Ambient AI documentation compared to control for NASA-TLX temporal demand	2	308	Nonrandomized studies	Serious^c^	Not serious	Not serious	Serious^e^	None	—	SMD 1.46 SD lower (2.81 lower to 0.11 lower)	⨁⨁◯◯ Low^c,e^
Ambient AI documentation compared to control for NASA-TLX effort	2	308	Nonrandomized studies	Serious^c^	Not serious	Not serious	Not serious^h^	None	—	SMD 1.29 SD lower (2.16 lower to 0.42 lower)	⨁⨁⨁◯ Moderate^c,h^
Ambient AI documentation compared to control for PFI^i^ work exhaustion	3	375	Nonrandomized studies	Serious^j^	Not serious	Not serious	Serious^k^	None	—	MD^l^ 0.35 lower (0.58 lower to 0.12 lower)	⨁⨁◯◯ Low^j,k^
Ambient AI documentation compared to control for burnout prevalence	3	502	Nonrandomized studies	Serious^j^	Not serious	Not serious	Not serious	Publication bias strongly suspected^m^	OR^n^ 0.47 (0.25 to 0.86)	180 fewer per 1000 (from 300 fewer to 38 fewer)	⨁⨁◯◯ Low^j,m^
Ambient AI documentation compared to control for documentation time	2	137	Nonrandomized studies	Serious^o^	Not serious	Not serious	Serious^e^	None	—	SMD 0.24 SD lower (1.1 lower to 0.61 higher)	⨁⨁◯◯ Low^e,o^

a AI: artificial intelligence.

b NASA-TLX: National Aeronautics and Space Administration Task Load Index.

c Observational pre-post designs.

d High *I*2.

e CI crosses null at k=2 under Knapp-Hartung.

f Not available.

g SMD: standardized mean difference.

h k=2.

i PFI: Professional Fulfillment Index.

j Predominantly observational pre-post designs.

k 95% prediction interval crosses null.

l MD: mean difference.

m Possible small-study effects/publication bias (small literature concentrated on two commercial products).

n OR: odds ratio.

o Observational designs.

The certainty for burnout reduction with ambient AI documentation was rated as low (⊕⊕◯◯). In total, 10 studies (2 RCTs and 8 observational) [4,7-10,38,39,42,44,45] reported burnout prevalence reductions of 7‐26 percentage points. Evidence was not downgraded for risk of bias, given RCT support. However, we noted possible small-study effects, given a small literature concentrated on 2 commercial products in which negative or null studies may be underrepresented [40].

The certainty for documentation time reduction with ambient AI documentation was rated as low (⊕⊕◯◯). In total, 8 studies (2 RCTs and 6 observational or quasi-experimental) [7,8,10,38,39,42,43,46] reported time savings of 0.9‐3 minutes per appointment. Evidence was downgraded for serious risk of bias (predominantly observational designs) and serious inconsistency, as effect sizes varied substantially across studies and settings.

The certainty regarding the effects of diagnostic imaging AI on cognitive workload was rated as very low (⊕◯◯◯). In total, 3 observational studies [16,47,48] showed inconsistent results ranging from no significant reduction to increased workload. Evidence was downgraded for serious risk of bias (all observational), serious inconsistency (conflicting directions of effect), and serious imprecision due to small sample sizes.

Similarly, the certainty for CDSS effects on cognitive workload was rated as very low (⊕◯◯◯). In total, 3 observational studies [17,49,50] showed highly variable results, with workload reductions of 7%‐50% in some studies but increases of 14 points in others (sepsis AI). Evidence was downgraded for very serious inconsistency (contradictory findings), serious indirectness (heterogeneous CDSS types and clinical contexts), and serious imprecision.

The certainty for AI-based burnout intervention was rated as low (⊕⊕◯◯). One RCT [51] demonstrated CBI reductions of 20‐23 points, but evidence was downgraded for serious imprecision due to reliance on a single study.

Taken together, the certainty of evidence for ambient AI documentation was limited both because the literature is commercially concentrated (Abridge and DAX Copilot dominate) and because the 95% PI for both burnout pools crosses the null effect line. Evidence for diagnostic imaging AI and CDSS remains very low certainty, with unintended workload increases observed in single studies of CADe imaging [16] and alert-based sepsis CDSS [17]. Documentation time benefits among ambient AI adopters carry low certainty (SMD −0.24, 95% CI −1.10 to 0.61).

### Synthesis of Findings

#### Ambient AI Documentation Systems

In total, 13 studies examined cognitive workload and burnout outcomes associated with ambient AI documentation tools [4,7-10,38,39,42-46,52], representing the largest evidence base in this review. These AI scribe systems convert patient-clinician conversations into draft clinical notes using speech recognition and natural language processing. The highest-quality evidence came from 2 RCTs. Lukac et al [7] conducted a 3-arm pragmatic RCT comparing 2 ambient AI scribes (DAX Copilot; Nuance Communications and Nabla; Nabla SAS) against usual care in 238 outpatient physicians across 14 specialties. Physicians randomized to AI scribes demonstrated significant reductions in task load, with DAX Copilot showing a 39.9-point reduction and Nabla showing a 31.7-point reduction on the Physician Task Load scale (*P*<.01). Work exhaustion also decreased significantly (−0.27 on a 0‐4 scale; *P*=.01). Documentation time decreased by 9.5% with Nabla (*P*=.02) but showed no significant change with DAX Copilot. Afshar et al [8] used a 24-week stepped-wedge individually randomized design with 66 health care practitioners, finding that Abridge (Abridge Inc) significantly reduced the work exhaustion and interpersonal disengagement composite score by 0.44 points (95% CI −0.62 to −0.25; *P*<.001) on the Stanford PFI.

Pre- and postimplementation studies demonstrated consistent findings. Olson et al [4] reported that burnout prevalence decreased from 51.9% to 38.8% (adjusted OR 0.26, 95% CI 0.13‐0.54; *P*<.001) and cognitive task load decreased by 2.64 points (*P*<.001) among 263 clinicians following Abridge implementation across 6 health systems. Shah et al [42] found that NASA-TLX scores decreased by 24.42 points (*P*<.001) and Stanford PFI burnout scores decreased by 1.94 points (*P*<.001) among 48 physicians using DAX Copilot. Hudson et al [43] demonstrated a 46.6% reduction in NASA-TLX composite scores (221.2 to 118.2; *P*<.001) in a randomized crossover study of 40 providers, with significant reductions across all subscales including mental demand (48.8% reduction), temporal demand (44.4% reduction), and effort (46.3% reduction).

The largest observational study by You et al [9] enrolled 1430 physicians and APPs across 2 academic medical centers; among the 265 Mass General Brigham clinicians who completed paired surveys at 42 days, burnout prevalence fell by 21.2 percentage points (50.6% to 29.4%; *χ*^2^_1_=42.4; *P*<.001), and well-being rose by 30.7 percentage points following ambient AI implementation over 42 days. Owens et al [44] found significantly lower OLBI disengagement scores in high versus low DAX users (16.3 vs 18.4; difference −2.1, 95% CI −3.8 to −0.4) among 110 primary care providers. Misurac et al [45] reported that burnout prevalence decreased from 69% to 43% (*P*=.005) among 35 providers. Stults et al [10] reported that documentation time decreased from 6.2 to 5.3 minutes per appointment (*P*<.001) and burnout prevalence from 42.1% to 35.1% among 100 ambulatory clinicians at Sutter Health using Abridge over 12 weeks. In this cohort, NASA-TLX mental, temporal, and effort subscales all decreased significantly. Duggan et al [39] found positive System Usability Scale scores and perceived reduction in documentation burden among 46 physicians and APPs using DAX Copilot over 8 weeks, though individual experiences were mixed.

Pelletier et al [38] provided the first pediatric ambient AI evidence. Documentation time decreased by 2.8 minutes per appointment (*P*<.001) with 1.5 hours saved weekly, alongside significant reductions in NASA-TLX workload and Mini-Z burnout scores among 84 pediatric physicians and APPs at Akron Children’s Hospital using Abridge over 6 months. The interrupted time series analysis design strengthened causal inference. A small simulation study by Bracken et al [46] examined Heidi Health (Heidi Health Pty Ltd) among 7 junior doctors in a simulated inpatient setting. The authors reported substantial reductions in documentation time (27 vs 128 seconds for progress notes; *P*<.0001) and NASA-TLX subscale reductions including a 79% reduction in frustration and an 81% reduction in effort, though the small sample size and simulation design limit generalizability. Similarly, Nawaz et al [52] conducted a crossover simulation study evaluating Lyrebird Health among 8 psychiatrists, demonstrating the largest effect size observed in this review, with NASA-TLX total workload scores of 25.0 with AI versus 461.3 without AI (MD −436.25; *P*<.001). Documentation quality also improved significantly on the Sheffield Assessment Instrument for Letters. However, the preprint status, small sample size (n=8), and simulation design warrant cautious interpretation.

The categories of diagnostic imaging AI, CDSS, and LLM-based tools were not eligible for meta-analytic pooling because of heterogeneity in study design, AI subtype, and outcome instrumentation. Study-level characteristics and key findings for these categories are reported in Tables 1, 3, and 4. Category-level interpretation is provided in the Discussion section.

#### AI-Based Burnout Intervention

One study examined AI not as a clinical tool but as a mechanism for delivering personalized burnout interventions. Baek and Cha [51] conducted a single-blind 3-group RCT among 120 nurses, comparing AI-tailored burnout interventions through a mobile app (Nurse Healing Space; Ewha Womans University) against standardized interventions and a waitlist control. The study achieved 0 dropouts. The AI-tailored group demonstrated significant reductions in CBI scores for client-related burnout (62.6 to 42.0; *F*=7.73; *P*=.001) and personal burnout (67.5 to 44.7; *F*=10.97; *P*<.0001) compared to controls. This study represents a distinct application of AI—using algorithmic personalization to address rather than potentially contribute to clinician burden.

#### Meta-Analysis Results

**Figure 3.**
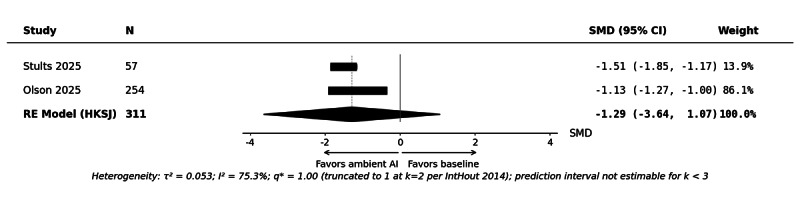
Forest plot of NASA-TLX mental demand subscale for ambient AI documentation versus baseline (k=2) [4,10]. Pooled SMD under the Knapp-Hartung-Sidik-Jonkman adjustment with REML estimation of τ^2^ (q* truncated to 1) was SMD −1.29 (95% CI −3.64 to 1.07); *I*^2^=75.3%. AI: artificial intelligence; HKSJ: Hartung-Knapp-Sidik-Jonkman; NASA-TLX: National Aeronautics and Space Administration Task Load Index; RE: random effect; REML: restricted maximum likelihood; SMD: standardized mean difference.

**Figure 4.**
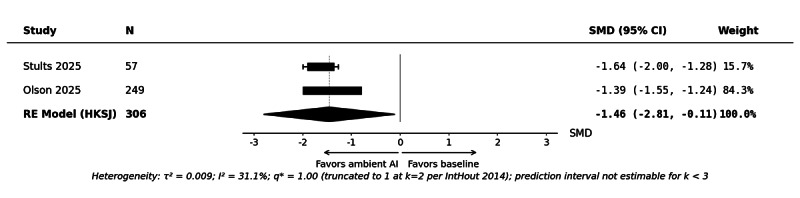
Forest plot of NASA-TLX temporal demand subscale for ambient AI documentation versus baseline (k=2) [4,10]. Knapp-Hartung-Sidik-Jonkman–adjusted pooled SMD −1.46 (95% CI −2.81 to −0.11); *I*^2^=31.1%. AI: artificial intelligence; HKSJ: Hartung-Knapp-Sidik-Jonkman; NASA-TLX: National Aeronautics and Space Administration Task Load Index; RE: random effect; SMD: standardized mean difference.

**Figure 5.**
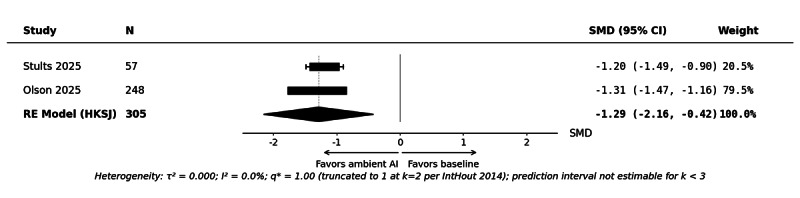
Forest plot of NASA-TLX effort subscale for ambient AI documentation versus baseline (k=2) [4,10]. Knapp-Hartung-Sidik-Jonkman–adjusted pooled SMD −1.29 (95% CI −2.16 to −0.42); *I*^2^=0%. AI: artificial intelligence; HKSJ: Hartung-Knapp-Sidik-Jonkman; NASA-TLX: National Aeronautics and Space Administration Task Load Index; RE: random effect; SMD: standardized mean difference.

**Figure 6.**
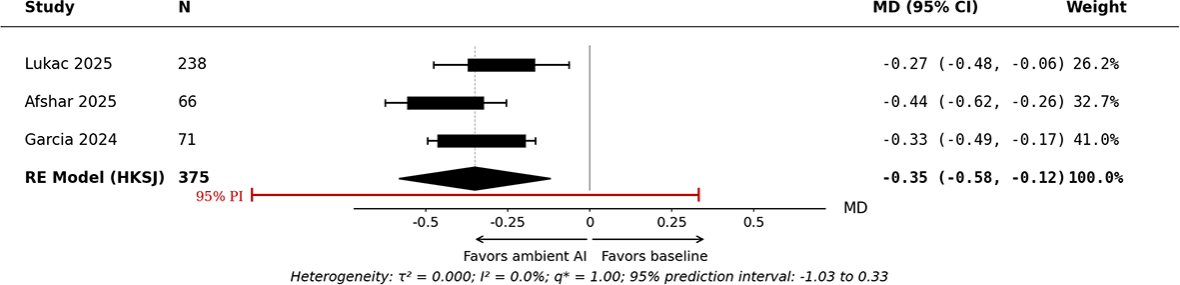
Forest plot of Professional Fulfillment Index work-exhaustion subscale for ambient AI documentation versus baseline (k=3; n=375) [7,8,37]. Knapp-Hartung-Sidik-Jonkman-adjusted pooled mean difference −0.35 (95% CI −0.58 to −0.12); *I*^2^=0%; 95% PI −1.03 to 0.33 (crosses null). AI: artificial intelligence; HKSJ: Hartung-Knapp-Sidik-Jonkman; MD: mean difference; PI: prediction interval; RE: random effect.

**Figure 7.**
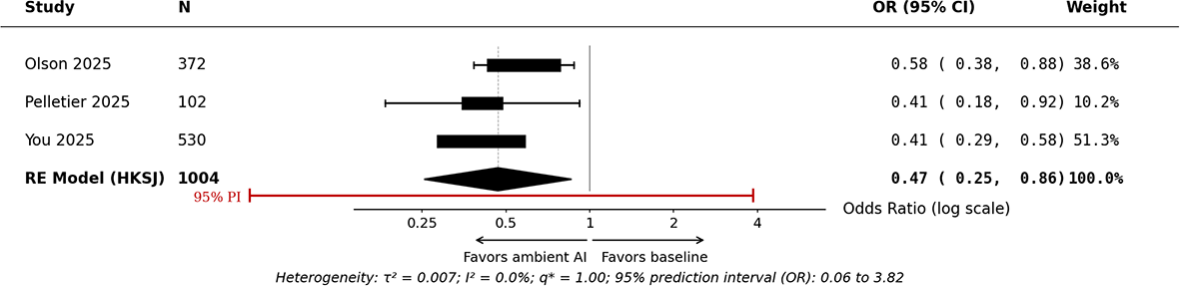
Forest plot of burnout prevalence (single-item or validated burnout instrument) for ambient AI documentation versus baseline (k=3; n=502) [4,9,38]. Knapp-Hartung-Sidik-Jonkman–adjusted pooled OR 0.47 (95% CI 0.25-0.86); *I*^2^=0%; 95% PI 0.06-3.82 (crosses null). AI: artificial intelligence; HKSJ: Hartung-Knapp-Sidik-Jonkman; OR: odds ratio; PI: prediction interval; RE: random effect.

**Figure 8.**
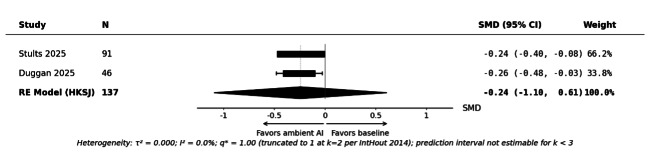
Forest plot of documentation time for ambient AI documentation versus baseline (k=2; n=137) [10,39]. Knapp-Hartung-Sidik-Jonkman–adjusted pooled SMD −0.24 (95% CI −1.10 to 0.61); *I*^2^=0%. AI: artificial intelligence; HKSJ: Hartung-Knapp-Sidik-Jonkman; RE: random effect; SMD: standardized mean difference.

Meta-analysis was conducted for ambient AI documentation studies with poolable data. All 6 prespecified pools were synthesized under the HKSJ adjustment with restricted maximum likelihood estimation of τ^2^ as the primary inferential framework, irrespective of k (with q* truncated to 1 per IntHout et al [34]). Forest plots for each pool are presented as Figures 3-8.

For NASA-TLX subscales (2 studies; n=305‐311) [4,10], SMDs were calculated due to different measurement scales (0‐20 vs 0‐10). When applying uniform HKSJ pooling at k=2, the subscales for mental and temporal demand yielded divergent findings regarding statistical significance. Mental demand did not achieve significance (SMD −1.291, 95% CI −3.645 to 1.068; *I*^2^=75.3%), whereas temporal demand demonstrated a significant reduction, as its CI excluded the null (SMD −1.458, 95% CI −2.808 to −0.109; *I*^2^=31.1%). The wide CI observed for mental demand reflects a combination of the conservative *t*-critical value at 1 degree of freedom and nontrivial between-study variance. Effort retained statistical significance with no detectable heterogeneity (SMD −1.291, 95% CI −2.160 to −0.421; *I*^2^=0%; q* truncated to 1). PIs were not estimable at k=2 (Figures 3–5).

For PFI work exhaustion (3 studies; n=375; [7,8,37]), the HKSJ-adjusted pooled MD was −0.350 (95% CI −0.582 to −0.119; *I*^2^=0%; τ^2^=0); the 95% PI was −1.034 to 0.333 and crossed the null effect line (Figure 6).

For burnout prevalence (3 studies; n=502; [4,9,38]), ambient AI implementation was associated with reduced odds of burnout: pooled OR was 0.470 (95% CI 0.254-0.861; *I*^2^=0%; τ^2^=0); the 95% PI was 0.059-3.817 and crossed the null effect line (Figure 7). You et al [9] contributed the largest burnout sample to this pool (n=265). Leave-one-out exclusion of You et al [9] preserved the direction of effect (Olson et al [4] showed a 13.1 percentage-point absolute reduction in burnout prevalence [51.9% → 38.8%]; Pelletier et al [38] showed a 21.6 percentage-point reduction [54.9% → 33.3%]), though pooled precision was reduced because of the resulting k=2.

For documentation time (2 studies; n=137) [10,39], the HKSJ-adjusted pooled SMD was −0.243 (95% CI −1.096 to 0.609; *I*^2^=0%; q* truncated to 1). The effect direction favored ambient AI but did not reach statistical significance under conservative pooling at k=2 (Figure 8). Sensitivity analyses varying the assumed pre-post correlation (*r*=0.5, 0.7, and 0.9) demonstrated stable point estimates across all outcomes; the width of CIs at k=2 was driven primarily by *t*-critical inflation rather than by the correlation assumption (Tables 5 and 6).

**Table 5.** Sensitivity of pooled effect estimates to the assumed pre-post correlation (*r*=0.5, 0.7, and 0.9) under uniform Hartung-Knapp-Sidik-Jonkman adjustment with restricted maximum likelihood estimation of τ^2^^a^.

Outcome (k studies) and *r*	Effect size (95% CI)	*I*^2^ (%)	τ^2b^
NASA-TLX^c^ mental demand (k=2)			
0.5	−1.28 (−3.62 to 1.06)	69.4	0.049
0.7^d^	−1.29 (−3.64 to 1.07)	75.3	0.053
0.9	−1.30 (−3.67 to 1.08)	81.2	0.058
NASA-TLX temporal demand (k=2)			
0.5	−1.45 (−2.71 to −0.18)	16.6	0.005
0.7^d^	−1.46 (−2.81 to −0.11)	31.1	0.009
0.9	−1.47 (−2.88 to −0.05)	45.6	0.013
NASA-TLX effort (k=2)			
0.5	−1.29 (−2.27 to −0.31)	0.0	0.000
0.7^d^	−1.29 (−2.16 to −0.42)	0.0	0.000
0.9	−1.29 (−2.03 to −0.55)	0.0	0.000
Documentation time (k=2)			
0.5	−0.24 (−1.33 to 0.85)	0.0	0.000
0.7^d^	−0.24 (−1.10 to 0.61)	0.0	0.000
0.9	−0.24 (−0.76 to 0.27)	0.0	0.000
Professional Fulfillment Index work exhaustion (k=3)			
N/A^e^	−0.35^f^ (−0.58 to −0.12)	0.0	0.000
Burnout prevalence (k=3)			
0.5^d^	0.47^g^ (0.25 to 0.86)	0.0	0.007
0.7	0.47^g^ (0.25 to 0.86)	0.0	0.007
0.9	0.47^g^ (0.25 to 0.86)	0.0	0.007

a q* was truncated to a minimum of 1 at k=2 [34]. For binary outcomes (burnout prevalence), log-odds ratios were computed directly from 2×2 tables; the pre-post correlation parameter is conventional and does not enter the variance calculation.

b τ2: between-study variance.

c NASA-TLX: National Aeronautics and Space Administration Task Load Index.

d Primary analysis.

e Not applicable. The work exhaustion pool was pooled using mean differences reported directly by the primary studies, so the assumed pre-post correlation did not enter the variance calculation.

f Mean difference.

g Odds ratio.

**Table 6.** Comparison of conventional random-effects pooling (*z*-based) versus uniform Hartung-Knapp-Sidik-Jonkman (HKSJ) pooling (*t*-based with q* truncated to 1) for the 4 k=2 prespecified pools (assumed pre-post correlation *r*=0.7)^a^.

Outcome (k=2) and method	SMD^b^ (95% CI)	CI width^c^	Ratio^d^
NASA-TLX^e^ mental demand (k=2)			
RE^f^ (*z*-based, no HKSJ)	−1.29 (−1.65 to −0.93)	0.73	1.0 (reference)
RE+uniform HKSJ	−1.29 (−3.64 to 1.07)	4.71	6.5×
NASA-TLX temporal demand (k=2)			
RE (*z*-based, no HKSJ)	−1.46 (−1.67 to −1.25)	0.42	1.0 (reference)
RE+uniform HKSJ	−1.46 (−2.81 to −0.11)	2.70	6.5×
NASA-TLX effort (k=2)			
RE (*z*-based, no HKSJ)	−1.29 (−1.42 to −1.16)	0.27	1.0 (reference)
RE+uniform HKSJ	−1.29 (−2.16 to −0.42)	1.74	6.5×
Documentation time (k=2)			
RE (*z*-based, no HKSJ)	−0.24 (−0.37 to −0.11)	0.26	1.0 (reference)
RE+uniform HKSJ	−0.24 (−1.10 to 0.61)	1.70	6.5×

a The width of the 95% CI under uniform HKSJ exceeds the conventional *z*-based interval by a factor of approximately 6.5 for all 4 k=2 pools; this reflects the *t*-critical value at 1 degree of freedom (*t*_₀.₀₂₅, ₁_=12.71) relative to the standard-normal critical value (*z*_₀.₀₂₅_=1.96). Point estimates are unchanged. This demonstrates that for the k=2 outcomes that did not reach significance under uniform HKSJ pooling, the width of the resulting CI was driven primarily by *t*-critical inflation rather than by between-study heterogeneity or the assumed pre-post correlation.

b SMD: standardized mean difference.

c CI width is computed as (upper bound−lower bound).

d Ratio is HKSJ CI width/*z*-based CI width; the constant factor of approximately 6.5 corresponds to *t*_₀.₀₂₅, ₁_/z_₀.₀₂₅_=12.706/1.960.

e NASA-TLX: National Aeronautics and Space Administration Task Load Index.

f RE: random effect.

We identified 3 key interpretive points regarding the statistical pooling. First, the uniform application of the HKSJ adjustment prioritizes protection against type I error inflation over narrow-band precision, particularly when unmodeled between-study variance is present [34]. Consequently, the pools for NASA-TLX mental demand and documentation time did not reach conventional statistical significance, despite their point estimates favoring ambient AI. Second, the 95% PIs for the 2 pools containing 3 studies (PFI work exhaustion and burnout prevalence) crossed the null effect line. This indicates that the true effect in a new, comparable setting could plausibly include no clinical benefit. Third, the NASA-TLX effort subscale was the only pool with 2 studies that retained statistical significance. This occurred because the estimated between-study variance was 0, which allowed the variance-inflation factor to be truncated to 1. Together, these findings reinforce the interpretation that the current pooled evidence base should be presented as preliminary rather than definitive, despite being suggestive of reductions in cognitive workload and burnout [35,40].

#### Summary of Effect Sizes

Across studies using the NASA-TLX or derivative instruments, effect sizes for AI documentation tools ranged from 14 to 40 points on the 100-point scale, representing moderate to large effects [4,7,8,10,38,39,42,43,46]. In total, 2 simulation studies reported substantially larger effects. Bracken et al [46] demonstrated 79%‐81% reductions in frustration and effort subscales, and Nawaz et al [52] reported an MD of 436.3 points on raw NASA-TLX total scores (25.0 vs 461.3; *P*<.001). Although this represents the largest effect size observed in this review, these findings require cautious interpretation, given the controlled simulation conditions and small sample sizes.

Burnout prevalence reductions ranged from 7 to 26 percentage points in absolute terms [4,9,10,45]. The randomized crossover study by Hudson et al [43] demonstrated the largest effect among real-world clinical studies with a 60.7% reduction in composite workload scores. In contrast, CDSS and diagnostic imaging AI showed smaller, inconsistent, or negative effects on cognitive workload [16,17,46,47]. Kandaswamy et al [17] found workload increased by 14 points with sepsis AI implementation, and Wenderott et al [16] found no workload reduction with prostate MRI AI despite increased reading time for complex cases.

Across the ambient AI documentation studies, the direction of effect was consistent. These tools were associated with reductions in cognitive workload and burnout, with the strongest evidence from the 2 RCTs [7,8]. Multisite studies were concordant with these findings across settings including pediatric [38] and psychiatric [52] specialties. However, diagnostic and alerting AI tools showed mixed effects highly dependent on implementation characteristics, alert frequency, case complexity, and workflow integration. Individual response variability was noted, with Duggan et al [39] reporting that not all clinicians benefited equally from ambient AI scribes. These findings suggest that the cognitive impact of AI varies substantially by application type, with documentation AI providing more consistent benefits than diagnostic or alerting AI systems.

## Discussion

### Principal Findings

This review set out to answer 3 questions, and our main findings map onto each. First, we asked how much AI changes the mental effort and strain that clinicians experience when it is built into their work, measuring this with well-established questionnaires. Combining the available studies, we found that AI used to automatically draft clinical notes (“ambient AI documentation”) was generally linked to less mental strain and less burnout. Of the 6 outcomes we evaluated, 4 showed a clear benefit. Two additional outcomes, mental demand and documentation time, also indicated potential benefits but lacked statistical conclusiveness due to the limited number of studies available for pooling. Second, we asked whether the effect depends on the type of AI, and it clearly did. Note-drafting AI tended to reduce workload and burnout, whereas AI systems designed to interpret medical images or issue decision-support alerts produced mixed results and, in some cases, unintended increases in clinician workload [16,17]. Third, we asked about subtler costs, including the effort required to double-check AI output and the tendency to overtrust it. None of the 21 studies measured these constructs directly with validated tools. We therefore discuss them as ideas to guide future research rather than as measured results.

This review brings validated workload questionnaires together with a conservative pooling method and PIs, which estimate how the effect might vary in new settings rather than only how precise the average is. Its main value lies in mapping the evidence across 21 studies (2885 health care workers in 7 countries, covering 5 kinds of AI: note-drafting tools, image-interpretation AI, on-screen decision-support alerts, LLM tools that draft inbox replies, and AI aimed at reducing burnout), and in grading how trustworthy the evidence is for each type of AI. The risk-of-bias assessments and the certainty-of-evidence summary are presented in Tables 2–4. Because only 2 to 3 studies could be combined for any single outcome, the pooled numbers are best read as support for this broader map of the evidence, not as conclusions on their own.

### Meta-Analyzed Outcomes: Ambient AI Documentation

Our findings should be interpreted within 3 constraints that bear on how much weight they can carry [35,36]. First, the benefits we observed for ambient AI documentation were not equally firm across outcomes. When the available studies were combined, the reductions in effort, work exhaustion, and burnout were the most consistent. In contrast, the apparent benefits for mental demand and documentation time rested on an insufficient number of studies to be considered established. Furthermore, the studies evaluating mental demand exhibited sufficient variability to preclude treating this reduction as a definitive finding [34]. Even for the outcomes that could be examined most fully, the average effect favored ambient AI, though its size in a new clinical setting could range from substantial to small.

A further and equally important point is that these results speak only to one kind of AI: every pooled outcome came from ambient AI documentation tools. AI as a category-wide remedy for clinician burnout is therefore not supported. The other technologies in this review, including diagnostic imaging AI, CDSS, the LLM inbox tool, and the AI burnout intervention, are discussed narratively and were not pooled. Second, most of the evidence comes from study designs that are more prone to bias. In total, 18 of the 21 studies were non-RCTs, with only 2 RCTs evaluating ambient AI [7,8]. Some of the most pronounced benefits were derived from very small simulation studies [46,52]. Because participants were largely volunteers and early adopters, the benefits may be overstated. Third, when we formally graded how trustworthy the evidence is using GRADE [41], only the reduction in cognitive workload with ambient AI documentation reached moderate confidence. Reductions in burnout and documentation time were graded as low confidence, and the evidence supporting diagnostic imaging AI, CDSS, and the single-trial AI burnout intervention was rated as very low confidence. Whether clinical AI improves the working life of the health care workforce overall (once accuracy, downstream patient safety, and long-term adaptation are taken into account) is not yet established.

### Comparison With Previous Literature

Our findings align with and extend the 2024 systematic review [15], which identified the absence of cognitive workload assessment as a notable gap in health care AI implementation research. While previous reviews focused primarily on AI diagnostic performance and efficiency metrics [15], our review specifically quantified the subjective cognitive experience of clinicians using validated instruments. The observed NASA-TLX reductions of 24‐40 points with ambient AI scribes exceed the minimally important difference threshold of 10 points suggested in human factors literature, indicating clinically meaningful cognitive burden relief [22].

The unintended workload increase observed with sepsis prediction AI [17] (NASA-TLX 43→57) corroborates the seminal work of Bainbridge [12] on the “ironies of automation,” predicting that systems designed to reduce human workload often create new cognitive demands through vigilance and oversight requirements. Similarly, the lack of workload reduction despite increased reading time with prostate MRI AI [16] supports the “out-of-the-loop” performance problem [13], where monitoring an automated system erodes the operator’s situation awareness and capacity to detect the very errors the automation was intended to prevent [14].

The direction of our pooled ambient-documentation findings is concordant with the larger primary literature that did not qualify for meta-analysis. Uncontrolled and pre-post evaluations of commercial ambient scribes have repeatedly reported reduced documentation burden, lower work exhaustion, and improved professional fulfillment [4,9,42,44,45]. Furthermore, a dedicated cognitive-load evaluation of an ambient platform reported reductions in subjective workload that align with the NASA-TLX effort effect observed in our synthesis [43]. These reports, however, share the structural features that constrain our pooled estimate. They are concentrated on a small number of commercial products, conducted in voluntary early-adopter cohorts, and predominantly use uncontrolled pre-post designs carrying a serious risk of bias under ROBINS-I [32]. Their convergence should therefore be read as consistent but low-certainty evidence rather than as confirmation of a robust effect, which is the reason the GRADE certainty for burnout reduction with ambient AI documentation was rated low rather than moderate [41].

The 2 RCTs available in this field [7,8] provide the least biased evidence; yet, both evaluated a single product over a short horizon, and neither measured downstream documentation accuracy or patient-safety end points. This scarcity of randomized data, combined with the small number of studies available for each outcome [34] and the fact that benefit could not be assumed in new settings even where the evidence was strongest [35], is the reason our synthesis stops short of asserting a generalizable benefit. The largest workload reductions originated from very small simulation studies [46,52], whose effect sizes are statistically fragile. This pattern suggests the potential presence of small-study effects [40] and reinforces the cautious interpretation demanded by the GRADE assessment.

### Studies Not Included in the Meta-Analysis: A Narrative Synthesis

In contrast to the ambient-documentation literature, evidence from diagnostic imaging AI and CDSS is heterogeneous and, in several reports, contrary to expectations. CADe for prostate MRI produced no workload reduction despite improved diagnostic performance [16], a pediatric sepsis prediction model increased perceived workload and alert fatigue [17], and a mobile CDSS tool improved guideline adherence without lowering mental workload [49]. Conversely, a knowledge-recommender embedded in radiology reporting reduced cognitive load [47]. This divergence is consistent with the imaging review that first identified the near-absence of workload measurement in this domain [15] and with automation-science predictions that alert- and flag-based systems impose monitoring and verification demands distinct from those of generative tools [12-14]. Because no 2 of these studies measured the same construct with the same instrument in the same setting, they could not be pooled, and the certainty of evidence for imaging AI and CDSS outcomes was correspondingly rated very low [41]. This inconsistency is best interpreted as genuine clinical and methodological heterogeneity rather than as evidence of no effect.

Two further categories of AI could likewise not be pooled and therefore described narratively. An LLM tool that drafts replies to patient inbox messages was evaluated in a single pre-post study, in which perceived task load fell substantially and work exhaustion improved over 5 weeks [37]. Because only one study examined this application, the finding is promising but cannot be generalized. It highlights a specific application of AI that differs fundamentally from both clinical note generation and image interpretation. Finally, one RCT examined AI not as a clinical tool but as a means of delivering a personalized burnout intervention. A smartphone app for nurses produced marked reductions in client-related and personal burnout relative to control over 4 weeks [51]. This study addresses clinician burnout from a different perspective, using AI to support well-being rather than to assist clinical work. Although its single-trial evidence was graded as low certainty, it illustrates that the role of AI in mitigating burnout extends beyond documentation tools.

### The Hidden Cost of AI: Rethinking Success Metrics

The AI categories included in this review differ fundamentally in function and in the clinician’s role with the tool. Generative AI tools, such as ambient AI scribes and LLM inbox-message drafts, produce clinician-facing draft text that the user reviews, edits, and approves. Conversely, discriminative AI tools, such as CDSS alerts and CADe diagnostic-imaging flags, push specific alerts that the clinician must verify against ground truth at the point of decision. The AI-based burnout intervention RCT delivers a psychological intervention outside the clinical workflow. We refer to the cognitive cost of reviewing, validating, and reconciling AI-generated outputs with clinical judgment as “verification burden.” As AI systems become ubiquitous, clinicians transition from content generation to content verification, which may explain the unintended workload increases observed with discriminative-AI tools. This framework, drawn from classical automation theory [12-14], is offered as hypothesis-generating. Direct measurement of these constructs represents a critical priority for future research.

A potentially important implication of this review, given the certainty caveats, is that AI implementation success cannot be reduced to diagnostic accuracy or time efficiency alone. Our finding that some AI systems unexpectedly increase cognitive workload despite improving efficiency challenges the prevailing assumption that “more AI equals better outcomes.” This shift toward supervisory review is a role that human cognitive architecture may be ill-suited to sustain [11,12]. This evidence suggests that regulatory bodies such as the Food and Drug Administration and Conformité Européenne marking authorities should consider incorporating human factors evaluation, including validated cognitive workload assessment, into the AI medical device approval process. Current regulatory frameworks focus predominantly on algorithmic performance metrics, potentially overlooking the real-world cognitive demands imposed on end users. Similarly, health care institutions implementing AI tools should routinely assess cognitive workload and burnout outcomes alongside traditional efficiency metrics [53,54].

The rapid adoption of ambient AI documentation reflects an ongoing transformation in clinical practice, as physician AI use doubled from 38% to 66% between 2023 and 2024 [1]. Our pooled estimates favor ambient AI documentation in early-adopter outpatient cohorts, but the size of this benefit may vary across settings, and the certainty of the evidence is low to moderate. Therefore, the present evidence supports cautious, evaluation-accompanied adoption rather than category-wide endorsement. Health care systems are increasingly viewing ambient AI scribes as one possible component of broader strategies for the clinician burnout crisis, although high-certainty evidence on the workforce-level impact of such adoption is not yet available [2].

### Strengths

This review has several strengths. We conducted a comprehensive search across 4 databases and included only studies that measured cognitive workload or burnout with validated instruments. We applied a rigorous risk of bias assessment using domain-appropriate tools, specifically RoB 2.0 for RCTs and ROBINS-I for non-RCTs, as detailed in Tables 2 and 3. Furthermore, we used a deliberately cautious approach to combining results across all outcomes to avoid overstating uncertain findings. We provided a clear grading of evidence certainty for each outcome, presented in Table 4, and included forest plots for every pool in Figures 3-8. Additional checks confirmed that our main conclusions did not depend on the specific analytical assumptions applied in the synthesis.

### Limitations

Several caveats apply to the interpretation of our findings. The positive evidence for ambient AI documentation rests on a narrow base. It is concentrated on 2 commercial products (Abridge and DAX Copilot), evaluated predominantly among volunteers and early adopters in US outpatient primary care settings, and followed for a short time (typically 8‐12 weeks). Because each outcome could be based on only a few studies, these pooled results are best read as preliminary rather than definitive, and the benefits for 2 of the 6 outcomes are no longer clear-cut once this limited evidence is taken into account. For this reason, we judged the certainty of the evidence for burnout reduction to be low rather than moderate, given the reliance on observational studies and a small literature dominated by 2 products with uniformly positive results. Throughout, claims are framed cautiously, with ambient AI documentation described as “associated with reductions in” cognitive workload and burnout rather than as reliably reducing them.

Finally, none of the 21 studies directly measured the subtler costs of working with AI. These unmeasured costs include the effort required to verify its output, the tendency to overtrust the system, and the subsequent loss of vigilance. As a result, the original question behind this review (whether AI genuinely reduces the mental burden of clinicians or simply shifts the demand from documentation to verification) cannot be answered with the present evidence and remains a priority for future research.

The following specific limitations should also be acknowledged. First, each outcome could be based on only a small number of studies, which limits how precisely the effects can be estimated, prevents the planned subgroup and related exploratory analyses [55], and leaves considerable uncertainty. Even for the 2 outcomes that could be examined most fully, work exhaustion and burnout prevalence, the average effect favored ambient AI, although the magnitude of this effect may differ in a new clinical setting. Second, the studies of some outcomes, most notably mental demand, varied considerably among themselves. This variability likely occurred because the studies used different measurement scales and were conducted across distinct clinical environments. Furthermore, combining results from abbreviated or modified versions of the same questionnaire adds further inconsistency [22,56,57]. Third, no study used the full MBI, even though it is the most thoroughly validated measure of burnout [25]. Fourth, we cannot rule out that smaller studies reported more favorable results than larger ones. Among the possible explanations, publication bias remains plausible because negative or null studies may be underrepresented in this rapidly commercializing field [40].

### Future Research Directions

The current evidence base, while suggestive of meaningful benefits from ambient AI documentation, remains preliminary, product-concentrated, and predominantly composed of short-term, single-institution evaluations conducted in early-adopter settings. Although ambient AI scribes appear to reduce documentation time and burnout reports, the present evidence does not allow us to verify documentation accuracy, downstream patient-safety end points, or net benefit once verification burden, automation bias, and long-term cognitive adaptation are accounted for.

Several research priorities must be addressed before strong clinical or policy recommendations can be formulated. First, future studies should use RCTs with preregistered protocols that pair clinician-reported workload and burnout outcomes with downstream patient-safety, documentation-accuracy, and diagnostic-error end points. Currently, only 4 of the 21 included studies used registered protocols. Second, prospective cohorts extending beyond 12 months are necessary to detect deskilling, adaptation, and longer-term cognitive effects. Third, researchers must prioritize the standardized measurement of verification burden, automation bias, automation complacency, and trust calibration using validated instruments. While narrower constructs such as trust, usability, and alert fatigue were assessed in some studies, the broader cognitive-cost framework remains unmeasured. Fourth, head-to-head comparative effectiveness studies across multiple commercial ambient AI scribe products, beyond early market leaders, are needed. Fifth, investigations should expand to non-US health care systems and specialties outside outpatient primary care, where workflow requirements and clinician burnout drivers may differ. Finally, future analyses should decompose the NASA-TLX into its specific subscales rather than relying on total scores, allowing for the precise identification of which cognitive domains AI affects most. Without such evidence, the workforce-level benefit of ambient AI scribes cannot yet be confirmed.

### Conclusions

This systematic review and meta-analysis is, to our knowledge, the first to combine validated cognitive-workload instruments with uniformly adjusted random-effects meta-analysis and PIs in human-AI interaction in health care. It is also the first to grade the certainty of evidence separately for 5 distinct AI categories: ambient documentation, diagnostic imaging AI, CDSS, LLM inbox tools, and AI-based burnout interventions. Whereas prior systematic reviews of health care AI have focused on diagnostic accuracy, efficiency, or implementation outcomes, the present synthesis quantifies the subjective cognitive experience and burnout of clinicians using validated instruments and grades the certainty of every quantitative outcome.

The contribution to the field is therefore a structured certainty-graded evidence base for the cognitive and burnout consequences of clinical AI. Ambient AI documentation is associated with reductions in NASA-TLX effort, PFI work exhaustion, and burnout prevalence in early-adopter cohorts; however, the evidence is still limited and uncertain. The observed benefits rest on a small number of studies, the effect sizes may not be uniform across settings, and technologies such as diagnostic imaging AI and CDSS have demonstrated context-dependent or unintended workload increases.

The real-world implications are immediate and concrete. Institutional pilots of ambient AI should be paired with prospective, validated-instrument measurement of cognitive workload and burnout. Regulatory human-factors review must be integrated alongside algorithmic-performance evaluation during AI medical-device approval. Furthermore, AI tool developers should incorporate human-centered design that anticipates verification burden as a primary outcome, a metric that was not directly measured in any included study. Until multiproduct, longer-term, multiregion evidence directly measures verification burden, automation bias, and downstream patient-safety end points, the net benefit of clinical AI on the health care workforce remains an open empirical question.

## Supplementary material

10.2196/93618 Multimedia Appendix 1 Search strategy.

10.2196/93618 Checklist 1 PRISMA-Abstract checklist.

10.2196/93618 Checklist 2 PRISMA 2020 checklist.

10.2196/93618 Checklist 3 PRISMA-S checklist.

## Abbreviations

AI: artificial intelligence
APP: advanced practice provider
CADe: computer-aided detection
CBI: Copenhagen Burnout Inventory
CDSS: clinical decision support system
GRADE: Grading of Recommendations Assessment, Development and Evaluation
HKSJ: Hartung-Knapp-Sidik-Jonkman
LLM: large language model
MBI: Maslach Burnout Inventory
MD: mean difference
MRI: magnetic resonance imaging
NASA-TLX: National Aeronautics and Space Administration Task Load Index
OLBI: Oldenburg Burnout Inventory
OR: odds ratio
PFI: Professional Fulfillment Index
PI: prediction interval
PRISMA: Preferred Reporting Items for Systematic Reviews and Meta-Analyses
PRISMA-S: Preferred Reporting Items for Systematic reviews and Meta-Analyses Literature Search Extension
RCT: randomized controlled trial
RoB 2: Risk of Bias tool version 2.0
ROBINS-I: Risk of Bias in Non-randomized Studies of Interventions
SMD: standardized mean difference

## References

[1] (2025). 2026 Physician survey on augmented intelligence. American Medical Association.

[2] Shanafelt TD, West CP, Sinsky C (2025). Changes in burnout and satisfaction with work-life integration in physicians and the general US working population between 2011 and 2023. Mayo Clin Proc.

[3] Sinsky C, Colligan L, Li L (2016). Allocation of physician time in ambulatory practice: a time and motion study in 4 specialties. Ann Intern Med.

[4] Olson KD, Meeker D, Troup M (2025). Use of ambient AI scribes to reduce administrative burden and professional burnout. JAMA Netw Open.

[5] Tierney AA, Gayre G, Hoberman B (2024). Ambient artificial intelligence scribes to alleviate the burden of clinical documentation. NEJM Catalyst.

[6] Albrecht M, Shanks D, Shah T (2025). Enhancing clinical documentation with ambient artificial intelligence: a quality improvement survey assessing clinician perspectives on work burden, burnout, and job satisfaction. JAMIA Open.

[7] Lukac PJ, Turner W, Vangala S (2025). Ambient AI scribes in clinical practice: a randomized trial. NEJM AI.

[8] Afshar M, Baumann MR, Resnik F (2025). A pragmatic randomized controlled trial of ambient artificial intelligence to improve health practitioner well-being. NEJM AI.

[9] You JG, Dbouk RH, Landman A (2025). Ambient documentation technology in clinician experience of documentation burden and burnout. JAMA Netw Open.

[10] Stults CD, Deng S, Martinez MC (2025). Evaluation of an ambient artificial intelligence documentation platform for clinicians. JAMA Netw Open.

[11] Oppenheimer DM (2008). The secret life of fluency. Trends Cogn Sci.

[12] Bainbridge L (1983). Analysis, Design and Evaluation of Man–Machine Systems.

[13] Endsley MR, Kiris EO (1995). The out-of-the-loop performance problem and level of control in automation. Hum Factors.

[14] Parasuraman R, Manzey DH (2010). Complacency and bias in human use of automation: an attentional integration. Hum Factors.

[15] Wenderott K, Krups J, Zaruchas F, Weigl M (2024). Effects of artificial intelligence implementation on efficiency in medical imaging-a systematic literature review and meta-analysis. NPJ Digit Med.

[16] Wenderott K, Krups J, Luetkens JA, Gambashidze N, Weigl M (2024). Prospective effects of an artificial intelligence-based computer-aided detection system for prostate imaging on routine workflow and radiologists’ outcomes. Eur J Radiol.

[17] Kandaswamy S, Muthu N, Braykov N (2025). Human performance evaluation of a pediatric artificial intelligence sepsis model. J Am Med Inform Assoc.

[18] West CP, Dyrbye LN, Shanafelt TD (2018). Physician burnout: contributors, consequences and solutions. J Intern Med.

[19] Page MJ, McKenzie JE, Bossuyt PM (2021). The PRISMA 2020 statement: an updated guideline for reporting systematic reviews. BMJ.

[20] Rethlefsen ML, Kirtley S, Waffenschmidt S (2021). PRISMA-S: an extension to the PRISMA Statement for Reporting Literature Searches in Systematic Reviews. Syst Rev.

[21] Eriksen MB, Frandsen TF (2018). The impact of patient, intervention, comparison, outcome (PICO) as a search strategy tool on literature search quality: a systematic review. J Med Libr Assoc.

[22] Hart SG, Staveland LE (1988). Development of NASA-TLX (Task Load Index): results of empirical and theoretical research. Adv Psychol.

[23] Reid GB, Nygren TE (1988). The subjective workload assessment technique: a scaling procedure for measuring mental workload. Adv Psychol.

[24] Paas F (1992). Training strategies for attaining transfer of problem-solving skill in statistics: a cognitive-load approach. J Educ Psychol.

[25] Maslach C, Jackson SE (1981). The measurement of experienced burnout. J Organ Behavior.

[26] Demerouti E, Bakker AB, Nachreiner F, Schaufeli WB (2001). The job demands-resources model of burnout. J Appl Psychol.

[27] Trockel M, Bohman B, Lesure E (2018). A brief instrument to assess both burnout and professional fulfillment in physicians: reliability and validity, including correlation with self-reported medical errors, in a sample of resident and practicing physicians. Acad Psychiatry.

[28] Kristensen TS, Borritz M, Villadsen E, Christensen KB (2005). The Copenhagen Burnout Inventory: a new tool for the assessment of burnout. Work Stress.

[29] Linzer M, Poplau S, Babbott S (2016). Worklife and wellness in academic general internal medicine: results from a national survey. J Gen Intern Med.

[30] Brooke J (1996). Usability Evaluation in Industry.

[31] Sterne JAC, Savović J, Page MJ (2019). RoB 2: a revised tool for assessing risk of bias in randomised trials. BMJ.

[32] Sterne JA, Hernán MA, Reeves BC (2016). ROBINS-I: a tool for assessing risk of bias in non-randomised studies of interventions. BMJ.

[33] Popay J, Roberts H, Sowden A Guidance on the conduct of narrative synthesis in systematic reviews. A product from the ESRC methods programme version 2006. https://www.york.ac.uk/media/crd/Guidance%20on%20the%20conduct%20of%20narrative%20synthesis%20in%20systematic%20review.pdf.

[34] IntHout J, Ioannidis JPA, Borm GF (2014). The Hartung-Knapp-Sidik-Jonkman method for random effects meta-analysis is straightforward and considerably outperforms the standard DerSimonian-Laird method. BMC Med Res Methodol.

[35] Borenstein M (2023). How to understand and report heterogeneity in a meta-analysis: the difference between I-squared and prediction intervals. Integr Med Res.

[36] Nagashima K, Noma H, Furukawa TA (2019). Prediction intervals for random-effects meta-analysis: a confidence distribution approach. Stat Methods Med Res.

[37] Garcia P, Ma SP, Shah S (2024). Artificial intelligence-generated draft replies to patient inbox messages. JAMA Netw Open.

[38] Pelletier JH, Watson K, Michel J, McGregor R, Rush SZ (2025). Effect of a generative artificial intelligence digital scribe on pediatric provider documentation time, cognitive burden, and burnout. JAMIA Open.

[39] Duggan MJ, Gervase J, Schoenbaum A (2025). Clinician experiences with ambient scribe technology to assist with documentation burden and efficiency. JAMA Netw Open.

[40] Sterne JAC, Sutton AJ, Ioannidis JPA (2011). Recommendations for examining and interpreting funnel plot asymmetry in meta-analyses of randomised controlled trials. BMJ.

[41] Guyatt GH, Oxman AD, Vist GE (2008). GRADE: an emerging consensus on rating quality of evidence and strength of recommendations. BMJ.

[42] Shah SJ, Devon-Sand A, Ma SP (2025). Ambient artificial intelligence scribes: physician burnout and perspectives on usability and documentation burden. J Am Med Inform Assoc.

[43] Hudson TJ, Albrecht M, Smith TR (2025). Impact of ambient artificial intelligence documentation on cognitive load. Mayo Clin Proc Digit Health.

[44] Owens LM, Wilda JJ, Hahn PY, Koehler T, Fletcher JJ (2024). The association between use of ambient voice technology documentation during primary care patient encounters, documentation burden, and provider burnout. Fam Pract.

[45] Misurac J, Knake LA, Blum JM (2025). The effect of ambient artificial intelligence notes on provider burnout. Appl Clin Inform.

[46] Bracken A, Babu AR, Whelehan S, Merghani K, Sheehan E, Feeley I (2026). Ambient AI reduces documentation time and enhances quality in a simulated inpatient setting. Surgeon.

[47] Lopez-Rippe J, Reddy M, Velez-Florez MC (2025). RADHawk—an AI-based knowledge recommender to support precision education, improve reporting productivity, and reduce cognitive load. Pediatr Radiol.

[48] Muzumala MG, Zulu EO, Chibuta P, Wu S, Shabestari B, Xing L Applications of Medical Artificial Intelligence AMAI 2024 Lecture Notes in Computer Science.

[49] Richardson KM, Fouquet SD, Kerns E, McCulloh RJ (2019). Impact of mobile device-based clinical decision support tool on guideline adherence and mental workload. Acad Pediatr.

[50] Sanderson BJ, Field JD, Kocaballi AB (2023). Clinical decision support versus a paper-based protocol for massive transfusion: impact on decision outcomes in a simulation study. Transfusion.

[51] Baek G, Cha C (2025). AI-assisted tailored intervention for nurse burnout: a three-group randomized controlled trial. Worldviews Evid Based Nurs.

[52] Nawaz FA, Bokhari SA, Usman FM (2025). Evaluating an ambient artificial intelligence scribe for documentation quality and efficiency in psychiatric consultations: a simulation-based study. medRxiv.

[53] Gong EJ, Bang CS (2026). Clinical implementation of artificial intelligence in endoscopy: a human-artificial intelligence interaction perspective. Korean J Gastroenterol.

[54] Gong EJ, Bang CS (2025). Artificial intelligence in colonoscopy: polyp fiction or clinical reality?. Clin Endosc.

[55] Higgins JPT, Thomas J, Chandler J (2024). Cochrane Handbook for Systematic Reviews of Interventions Version 65 (Updated August 2024).

[56] Chen ELY, Li JW (2025). Computer-aided quality control in colonoscopy: clinical applications and limitations. Clin Endosc.

[57] Lwin WP, Ichimasa K, Kudo SE (2025). Clinical significance of computer-aided quality assessment systems in colonoscopy: a comprehensive review. Clin Endosc.

