# Supplementary material for: Cognitive Workload and Mental Burden in Health Care Professionals Interacting With AI: Systematic Review and Meta-Analysis
Source: J Med Internet Res. 2026 Aug 4;28:e93618. doi: 10.2196/93618 (PMC13437520; doi:10.2196/93618)
Supplement: Supplementary file 1 [file jmir-v28-e93618-s001.docx]

**Table S1. Search strategies**

Search strategies for four electronic databases (search date: January 2026; earliest year limit: 2015). The search combined three concepts: (1) AI/clinical AI tools, (2) healthcare professionals, and (3) cognitive workload/burnout outcomes.

| **Database** | **Platform/Interface** | **Search Date** | **Results** |
| --- | --- | --- | --- |
| MEDLINE (PubMed) | NLM | January 2026 | 2055 |
| EMBASE | OVID | January 2026 | 6024 |
| Cochrane CENTRAL | Cochrane Library | January 2026 | 65 |
| Web of Science | Clarivate | January 2026 | 704 |

**1. MEDLINE (PubMed)**

**Search query: #1 AND #2 AND #3: 2055**

#1 Artificial Intelligence Concept: 471121

("artificial intelligence"[MeSH Terms] OR "machine learning"[MeSH Terms] OR "deep learning"[MeSH Terms] OR "neural networks, computer"[MeSH Terms] OR "natural language processing"[MeSH Terms] OR "artificial intelligence"[Title/Abstract] OR "machine learning"[Title/Abstract] OR "deep learning"[Title/Abstract] OR "clinical decision support"[Title/Abstract] OR "AI-assisted"[Title/Abstract] OR "AI-powered"[Title/Abstract] OR "ambient scribe*"[Title/Abstract] OR "AI scribe*"[Title/Abstract] OR "large language model*"[Title/Abstract] OR "LLM"[Title/Abstract] OR "GPT"[Title/Abstract] OR "ChatGPT"[Title/Abstract] OR "generative AI"[Title/Abstract] OR "computer-aided detection"[Title/Abstract] OR "computer-aided diagnosis"[Title/Abstract] OR "CAD system*"[Title/Abstract] OR "clinical decision support system*"[Title/Abstract] OR "CDSS"[Title/Abstract] OR "predictive analytics"[Title/Abstract] OR "diagnostic AI"[Title/Abstract])

#2 Healthcare Professionals Concept: 2238970

("health personnel"[MeSH Terms] OR "physicians"[MeSH Terms] OR "nurses"[MeSH Terms] OR "radiologists"[Title/Abstract] OR "pathologists"[Title/Abstract] OR "clinician*"[Title/Abstract] OR "physician*"[Title/Abstract] OR "doctor*"[Title/Abstract] OR "nurse*"[Title/Abstract] OR "healthcare professional*"[Title/Abstract] OR "health care professional*"[Title/Abstract] OR "healthcare worker*"[Title/Abstract] OR "medical staff"[Title/Abstract] OR "resident*"[Title/Abstract] OR "trainee*"[Title/Abstract] OR "hospitalist*"[Title/Abstract] OR "practitioner*"[Title/Abstract])

#3 Cognitive Workload/Burden Concept: 107865

("workload"[MeSH Terms] OR "burnout, professional"[MeSH Terms] OR "mental fatigue"[MeSH Terms] OR "cognitive load"[Title/Abstract] OR "cognitive workload"[Title/Abstract] OR "mental workload"[Title/Abstract] OR "mental burden"[Title/Abstract] OR "cognitive burden"[Title/Abstract] OR "cognitive fatigue"[Title/Abstract] OR "mental fatigue"[Title/Abstract] OR "NASA-TLX"[Title/Abstract] OR "task load index"[Title/Abstract] OR "burnout"[Title/Abstract] OR "professional burnout"[Title/Abstract] OR "work exhaustion"[Title/Abstract] OR "alert fatigue"[Title/Abstract] OR "alarm fatigue"[Title/Abstract] OR "automation bias"[Title/Abstract] OR "automation complacency"[Title/Abstract] OR "verification burden"[Title/Abstract] OR "human factors"[Title/Abstract] OR "usability"[Title/Abstract])

**2. EMBASE (via OVID)**

**Search query: Line 12 AND Line 23 AND Line 38: 6024**

**Artificial Intelligence Concept: 603840**

1. exp artificial intelligence/

2. exp machine learning/

3. exp deep learning/

4. exp natural language processing/

5. exp clinical decision support system/

6. exp computer assisted diagnosis/

7. (artificial intelligence or machine learning or deep learning).tw.

8. (clinical decision support or CDSS or CAD system*).tw.

9. (ambient scribe* or AI scribe* or AI-assisted or AI-powered).tw.

10. (large language model* or LLM or GPT or ChatGPT or generative AI).tw.

11. (diagnostic AI or predictive analytics or computer aided).tw.

12. 1 or 2 or 3 or 4 or 5 or 6 or 7 or 8 or 9 or 10 or 11 [combines lines 1–11; AI concept set]

**Healthcare Professionals Concept: 3599704**

13. exp health care personnel/

14. exp physician/

15. exp nurse/

16. exp radiologist/

17. exp pathologist/

18. (clinician* or physician* or doctor* or nurse*).tw.

19. (healthcare professional* or health care professional*).tw.

20. (healthcare worker* or medical staff).tw.

21. (radiologist* or pathologist*).tw.

22. (resident* or trainee* or hospitalist* or practitioner*).tw.

23. 13 or 14 or 15 or 16 or 17 or 18 or 19 or 20 or 21 or 22 [combines lines 13–22; healthcare-professionals concept set]

**Cognitive Workload/Burden Concept: 2689269**

24. exp workload/

25. exp burnout/

26. exp mental fatigue/

27. exp cognitive load/

28. exp human factors engineering/

29. (cognitive workload or cognitive load or mental workload).tw.

30. (mental burden or cognitive burden or psychological burden).tw.

31. (cognitive fatigue or mental fatigue).tw.

32. (NASA-TLX or task load index or workload assessment).tw.

33. (burnout or professional burnout or work exhaustion).tw.

34. (alert fatigue or alarm fatigue).tw.

35. (automation bias or automation complacency).tw.

36. (verification burden or human factors or usability).tw.

37. (Maslach Burnout Inventory or MBI or Professional Fulfillment Index).tw.

38. 24 or 25 or 26 or 27 or 28 or 29 or 30 or 31 or 32 or 33 or 34 or 35 or 36 or 37 [combines lines 24–37; cognitive-workload/burnout concept set; line 38 is NOT self-referential]

**3. Cochrane CENTRAL**

**Search query: #4 AND #8 AND #13: 65**

#1 MeSH descriptor: [Artificial Intelligence] explode all trees

#2 MeSH descriptor: [Machine Learning] explode all trees

#3 ("artificial intelligence" OR "machine learning" OR "deep learning" OR "clinical decision support" OR "ambient scribe" OR "AI scribe" OR "large language model" OR "LLM" OR "GPT" OR "ChatGPT" OR "CAD system" OR "computer aided"):ti,ab,kw

#4 #1 OR #2 OR #3 [combines lines #1–#3; AI concept set]: 13208

#5 MeSH descriptor: [Health Personnel] explode all trees

#6 MeSH descriptor: [Physicians] explode all trees

#7 (clinician* OR physician* OR doctor* OR nurse* OR radiologist* OR pathologist* OR "healthcare professional" OR "healthcare worker" OR practitioner*):ti,ab,kw

#8 #5 OR #6 OR #7 [combines lines #5–#7; healthcare-professionals concept set]: 16641

#9 MeSH descriptor: [Workload] explode all trees

#10 MeSH descriptor: [Burnout, Professional] explode all trees

#11 ("cognitive workload" OR "cognitive load" OR "mental workload" OR "mental burden" OR "cognitive burden" OR "mental fatigue" OR "cognitive fatigue"):ti,ab,kw

#12 ("NASA-TLX" OR "task load index" OR burnout OR "alert fatigue" OR "automation bias" OR "human factors"):ti,ab,kw

#13 #9 OR #10 OR #11 OR #12 [combines lines #9–#12; cognitive-workload/burnout concept set]: 5327

#14 #4 AND #8 AND #13

**4. Web of Science**

**Search query: #1 AND #2 AND #3: 704**

#1 TS=("artificial intelligence" OR "machine learning" OR "deep learning" OR "clinical decision support" OR "CDSS" OR "ambient scribe*" OR "AI scribe*" OR "large language model*" OR "LLM" OR "GPT" OR "ChatGPT" OR "generative AI" OR "computer aided diagnosis" OR "CAD system*" OR "diagnostic AI" OR "predictive analytics")

#2 TS=(clinician* OR physician* OR doctor* OR nurse* OR radiologist* OR pathologist* OR "healthcare professional*" OR "health care professional*" OR "healthcare worker*" OR "medical staff" OR resident* OR practitioner*)

#3 TS=("cognitive workload" OR "cognitive load" OR "mental workload" OR "mental burden" OR "cognitive burden" OR "mental fatigue" OR "cognitive fatigue" OR "NASA-TLX" OR "task load index" OR burnout OR "professional burnout" OR "alert fatigue" OR "alarm fatigue" OR "automation bias" OR "automation complacency" OR "verification burden" OR "human factors")

#4 #1 AND #2 AND #3

**5. Grey literature and additional sources**

**Reference list searching:** Reference lists of all included studies and relevant systematic reviews were manually searched for additional eligible studies.

**Forward citation tracking:** Google Scholar was used to identify studies citing the included articles.

**Preprint servers:** medRxiv and arXiv were searched using combinations of key terms (AI, healthcare, cognitive workload, burnout).
