# Supplementary material for: Cognitive Workload and Mental Burden in Health Care Professionals Interacting With AI: Systematic Review and Meta-Analysis
Source: J Med Internet Res. 2026 Aug 4;28:e93618. doi: 10.2196/93618 (PMC13437520; doi:10.2196/93618)
Supplement: Supplementary file 4 [file jmir-v28-e93618-s004.docx]

**PRISMA-S Checklist**

| **Section/topic** | **#** | **Checklist item** | **Location(s) Reported** |
| --- | --- | --- | --- |
| **INFORMATION SOURCES AND METHODS** | | | |
| Database name | 1 | Name each individual database searched, stating the platform for each. | Methods, Information sources, p.11; Multimedia Appendix 4, Table S1 (MEDLINE/PubMed, EMBASE/OVID, Cochrane CENTRAL, Web of Science) |
| Multi-database searching | 2 | If databases were searched simultaneously on a single platform, state the name of the platform, listing all of the databases searched. | Multimedia Appendix 4, Table S1 (databases searched separately on respective native platforms) |
| Study registries | 3 | List any study registries searched. | Methods, Reporting standards and protocol deviations, p.15-17 (study registries [e.g., ClinicalTrials.gov] not searched; rationale provided) |
| Online resources and browsing | 4 | Describe any online or print source purposefully searched or browsed (e.g., tables of contents, print conference proceedings, web sites), and how this was done. | Methods, Reporting standards and protocol deviations, p.15-17 (preprint servers medRxiv and arXiv screened) |
| Citation searching | 5 | Indicate whether cited references or citing references were examined, and describe any methods used for locating cited/citing references (e.g., browsing reference lists, using a citation index, setting up email alerts for references citing included studies). | Methods, Search strategy, p.11 (forward and backward citation tracking of included studies and key prior reviews) |
| Contacts | 6 | Indicate whether additional studies or data were sought by contacting authors, experts, manufacturers, or others. | Methods, Reporting standards and protocol deviations, p.15-17 (formal expert/author contact limited to clarification of published study data) |
| Other methods | 7 | Describe any additional information sources or search methods used. | Methods, Search strategy, p.11 (grey-literature screening; reference-list checking) |
| **SEARCH STRATEGIES** | | | |
| Full search strategies | 8 | Include the search strategies for each database and information source, copied and pasted exactly as run. | Multimedia Appendix 4, Table S1 (full Boolean search strings for MEDLINE, EMBASE, Cochrane CENTRAL, Web of Science) |
| Limits and restrictions | 9 | Specify that no limits were used, or describe any limits or restrictions applied to a search (e.g., date or time period, language, study design) and provide justification for their use. | Multimedia Appendix 4, Table S1 (date limits and language restrictions specified per database) |
| Search filters | 10 | Indicate whether published search filters were used (as originally designed or modified), and if so, cite the filter(s) used. | Methods, Reporting standards and protocol deviations, p.15-17 (no methodological filters applied; review intentionally included multiple study designs) |
| Prior work | 11 | Indicate when search strategies from other literature reviews were adapted or reused for a substantive part or all of the search, citing the previous review(s). | Methods, Reporting standards and protocol deviations, p.15-17 (search strategy not derived from a prior published search) |
| Updates | 12 | Report the methods used to update the search(es) (e.g., rerunning searches, email alerts). | Methods, Reporting standards and protocol deviations, p.15-17 (search not re-run after January 2026 cutoff) |
| Dates of searches | 13 | For each search strategy, provide the date when the last search occurred. | Methods, Information sources, p.11; Multimedia Appendix 4, Table S1 (each database last searched January 2026) |
| **PEER REVIEW** | | | |
| Peer review | 14 | Describe any search peer review process. | Methods, Reporting standards and protocol deviations, p.15-17 (search strategy not formally peer-reviewed by an independent information specialist; developed iteratively by two reviewer-authors and refined through pilot searches and reference-list checking) |
| **MANAGING RECORDS** | | | |
| Total Records | 15 | Document the total number of records identified from each database and other information sources. | Multimedia Appendix 4, Table S1; Results, Study Selection, p.18; Figure 1 (8,848 records: PubMed/MEDLINE n=2,055; EMBASE n=6,024; Cochrane CENTRAL n=65; Web of Science n=704) |
| Deduplication | 16 | Describe the processes and any software used to deduplicate records from multiple database searches and other information sources. | Methods, Selection process, p.11; Results, Study Selection, p.18 (EndNote v.21; 1,520 duplicates removed) |
|  |  |  |  |
| PRISMA-S: An Extension to the PRISMA Statement for Reporting Literature Searches in Systematic Reviews | | |  |
| Rethlefsen ML, Kirtley S, Waffenschmidt S, Ayala AP, Moher D, Page MJ, Koffel JB, PRISMA-S Group. | | |  |
| Last updated February 27, 2020. | |  |  |
